# Activity of Single Insect Olfactory Receptors Triggered by Airborne Compounds Recorded in Self-Assembled Tethered Lipid Bilayer Nanoarchitectures

**DOI:** 10.1021/acsami.3c09304

**Published:** 2023-09-27

**Authors:** David Kleinheinz, Chiara D’Onofrio, Colm Carraher, Anil Bozdogan, Ulrich Ramach, Bernhard Schuster, Manuela Geiß, Markus Valtiner, Wolfgang Knoll, Jakob Andersson

**Affiliations:** † Austrian Institute of Technology GmbH, Giefinggasse 4, Vienna 1210, Austria; ‡ The New Zealand Institute for Plant and Food Research, 120 Mount Albert Road, Sandringham, Auckland 1025, New Zealand; § Technische Universität Wien, Wiedner Hauptstr. 8-10/134, Wien 1040, Austria; ∥ CEST Kompetenzzentrum für Oberflächentechnologie, Viktor Kaplan-Straße 2, Wiener Neustadt 2700, Austria; ⊥ Department of Bionanosciences, Institute of Synthetic Bioarchitectures, University of Natural Resources and Life Sciences (BOKU), Muthgasse 11, Vienna 1190, Austria; # Software Competence Center Hagenberg GmbH, Softwarepark 32a, Hagenberg 4232, Austria; ¶ Danube Private University, Steiner Landstraße 124, Krems an der Donau 3500, Austria

**Keywords:** bioelectronic nose, lipid bilayer, membrane proteins, ion channels, insect olfaction, *Drosophila melanogaster*

## Abstract

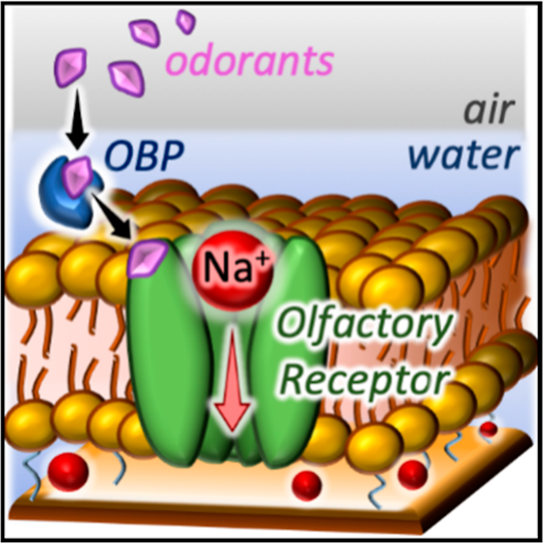

Membrane proteins are among the most difficult to study as they are embedded in the cellular membrane, a complex and fragile environment with limited experimental accessibility. To study membrane proteins outside of these environments, model systems are required that replicate the fundamental properties of the cellular membrane without its complexity. We show here a self-assembled lipid bilayer nanoarchitecture on a solid support that is stable for several days at room temperature and allows the measurement of insect olfactory receptors at the single-channel level. Using an odorant binding protein, we capture airborne ligands and transfer them to an olfactory receptor from *Drosophila melanogaster* (OR22a) complex embedded in the lipid membrane, reproducing the complete olfaction process in which a ligand is captured from air and transported across an aqueous reservoir by an odorant binding protein and finally triggers a ligand-gated ion channel embedded in a lipid bilayer, providing direct evidence for ligand capture and olfactory receptor triggering facilitated by odorant binding proteins. This model system presents a significantly more user-friendly and robust platform to exploit the extraordinary sensitivity of insect olfaction for biosensing. At the same time, the platform offers a new opportunity for label-free studies of the olfactory signaling pathways of insects, which still have many unanswered questions.

## 1 Introduction

**Figure 1:**
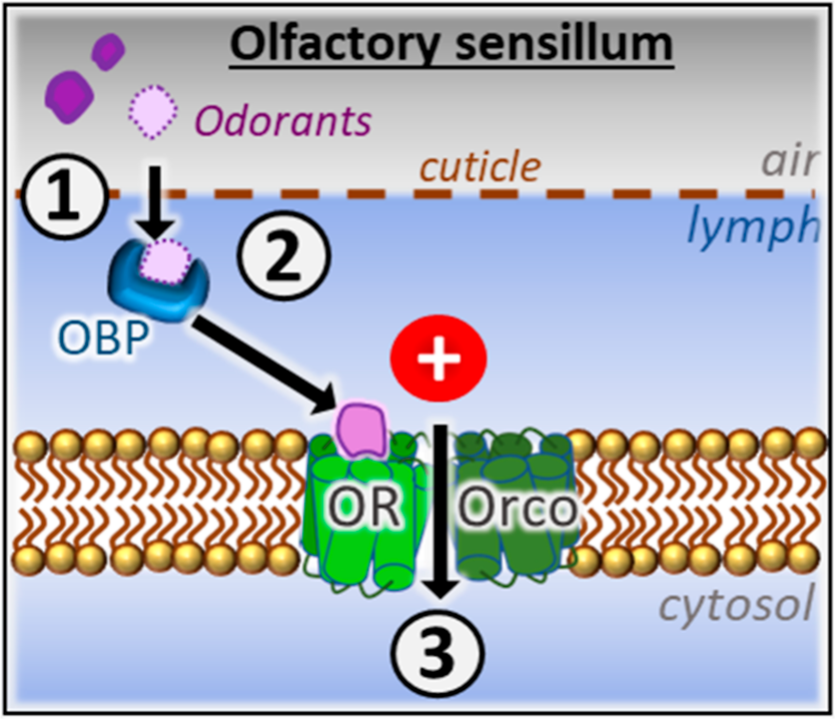
Insect olfaction process. (1) Binding of the odorant to an OBP, (2) release of the odorant from the OBP to the OR, and (3) opening of the ion channel in the Orco of the OR/Orco complex.

The insect life cycle revolves predominantly around the perception of small concentrations of volatile organic compounds (VOCs). Olfactory cues are used to locate food sources and mating partners and to avoid predators. Unlike vertebrates, insects do not rely on G protein-coupled receptors and instead use a heteromeric ligand-gated receptor complex comprising the odorant receptor coreceptor (Orco, acting as an ion channel) and an odorant receptor (OR, conferring ligand selectivity).^1,2^ See Figure 1 for a schematic overview of the insect olfaction process. The Orco subunit is highly conserved across insect species and does not respond to odorants on its own.^3^ Orco has been shown to be triggered only by synthetic ligands, such as the compound VUAA1.^4^ ORs, on the other hand, vary widely in their protein sequences and cannot act as ion channels without Orco, except in the case of some insects that lack an Orco gene—for example, *Machilis hrabei*,^5^ instead appearing to be responsible for ligand selectivity.^2,4,6^

Odorant binding proteins (OBPs) are thought to act as a shuttle to transfer hydrophobic odorant molecules across the aqueous lymph to the receptors in the neuronal membrane, although they also appear to fulfill other roles, such as filtering which odorants are detected.^7^ It has been shown, however, that OBPs may not be necessary to transport all odorant molecules, as even in the absence of OBPs, olfactory receptors can still respond to some ligands.^8^ It is possible that rather than transporting the pheromone 11-*cis*-vaccenyl acetate to an OR, the holo- (or ligand-bearing) form of DmelOBP76a (also known as LUSH) triggers the olfactory receptor.^9,10^ Suppressing the expression of some OBPs was found to result in an increased response to some compounds.^7^ It is therefore clear that only determining the ligand affinities of an OBP is insufficient to understand its role in the olfaction process. A direct method to observe the signalling pathway is needed, as suppressing the expression of selected OBPs and measuring antennal response to an odorant is a highly complex process and does not eliminate the possibility that the expression of other OBPs is upregulated in order to compensate.

This, and the discovery that OBPs are expressed in tissues other than olfactory sensilla^11^ indicate that they may be involved in other processes besides the transport of odorants, such as buffering of sudden changes in odorant concentrations or preventing behaviorally irrelevant VOCs from triggering the receptor.^12^ Many open questions remain regarding the olfactory process, particularly the mechanism behind the transfer of the ligand to the receptor and whether OBPs are required for this and receptor activation.^13^ What triggers the release of the ligand from the OBP to the receptor is unclear, as is the process by which the receptor is reset and whether this also involves OBPs. However, the olfaction process takes place in the cellular membrane, which is a complex and fragile environment with limited experimental accessibility. As a result, the study of insect olfaction currently relies on complex, time-intensive techniques such as sensillum electrophysiology, cell-based assays,^14,15^ and behavioral studies with genetically altered organisms.^5,16–19^

Since it has recently become possible to produce odorant receptors recombinantly,^20–23^ synthetic olfactory receptors can now be used for “bioelectronic nose” devices.^24–26^ However, while these devices have achieved excellent sensitivity, they are designed primarily to generate a signal and not to investigate the underlying biophysical mechanisms. In one example, background currents in the pA-range were possible, but the noise remained too high for the measurement of single-channel activity.^24^ We present here a platform that is accessible to a wide range of experimental techniques while also allowing quantification of receptor activity at the single-channel level. It can thus be used not only at a fundamental biophysical level but also as a new, highly sensitive, and robust biosensing platform.

### 1.1 Model Systems to Study Insect Olfaction

One of the most common model systems for electrophysiological studies of ion channels are free-standing bilayer lipid membranes or black lipid membranes (BLMs) which have been optimized for multiplexed ion channel studies.^27^ However, while they provide an excellent environment for single-channel physiology, BLMs are highly susceptible to mechanical and thermal disturbances and have low stability^28^ (although this can be improved by using nanoporous rather than μm-sized apertures).^29^

In addition, BLMs are suitable only for electrical measurements and some optical techniques and do not allow other techniques such as atomic force microscopy (AFM), neutron scattering, or surface plasmon resonance (SPR) to be used. AFM in particular is a highly attractive technique as recent advances now allow protein structures to be determined down to the level of single amino acids.^30^

**Figure 2:**
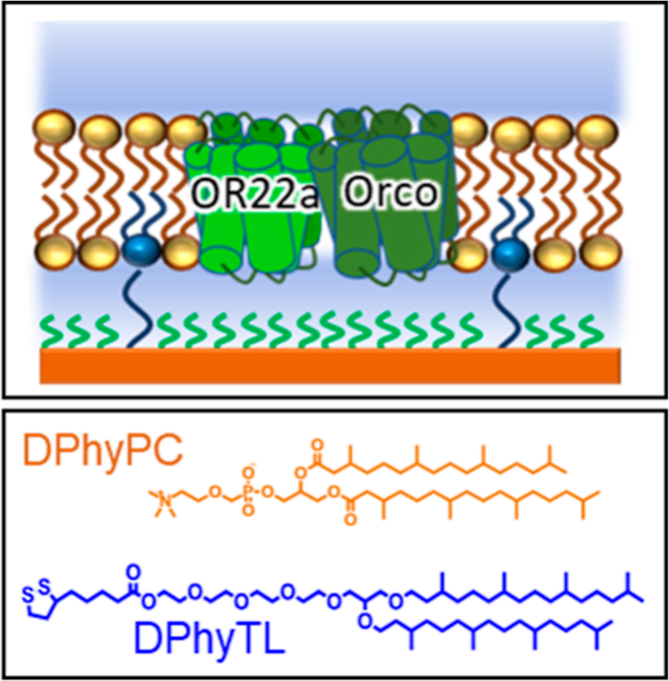
stBLM architecture containing the DmOR22a/Orco complex, composed of the anchorlipid 2,3-di-O-phytanyl-*sn*-glycerol-1-tetraethylene glycol-d,l-α-lipoic acid ester lipid (DPhyTL, blue) and 1,2-diphytanoyl-*sn*-glycero phosphocholine (DPhyPC, orange). The anchorlipid is diluted with mercaptoethanol (green).

By depositing the lipid bilayer on a supporting material such as glass, gold, or silicon, their stability can be increased but the inner leaflet interacts strongly with the substrate, and there is limited space for protein incorporation, particularly proteins with large submembrane domains such as insect ORs.^16^ To reduce membrane interactions with the substrates, tethered membrane systems can be used where the lipid bilayer is suspended above the supporting material using anchorlipids containing a spacer molecule such as tetra(ethylene glycol) in the case of DPhyTL, see Figure 2.^31–34^

While DPhyTL-based lipid bilayers can remain intact for several months, the tightly packed inner membrane leaflet and spacer region contains only 5% water,^35^ making them poorly suited for ion channel incorporation. The submembrane space can be increased by diluting the anchorlipid with a spacer compound such as mercaptoethanol, creating a sparsely tethered bimolecular lipid membrane (stBLM, see Figure 2).^36^ However, the submembrane reservoir in this configuration is still restricted in height to approximately 2 nm, which is insufficient to accommodate Orco, which extends up to 4 nm below the membrane.^16^ While lipid mobility is decreased by approximately 50% in tethered membranes^37^ compared to free-standing lipid bilayers, the olfactory receptor requires only sufficient fluidity to open and close as it retains its function even when completely immobilized.^22^

The submembrane reservoir could be increased by extending the length of the spacer segment; however, it has been shown that this also leads to significantly increased defect density (resulting in higher background currents) and is thus not well-suited to study ion channels.^35,36^ Instead, it was necessary to slightly increase substrate roughness and optimize the tethering density (ratio between anchorlipid and spacer) to accommodate Orco.^25^ We show here that the membrane architecture containing Orco can also be used to functionally reconstitute the complete complex of the olfactory receptor OR22a and olfactory receptor coreceptor Orco from the fruit fly (*Drosophila melanogaster*) and record its function at the single-channel level triggered by introducing airborne compounds. Moreover, the flexibility and robustness of the system also allowed us to examine the membrane systems with high-speed liquid AFM, SPR, and surface plasmon fluorescence spectroscopy (SPFS).

## 2 Materials and Methods

### 2.1 Chemicals

Fresh ultrapure water obtained from the Sartorius Arium Pro system (18.2 MΩ cm resistance) was used for all experiments. Spectroscopic-grade methanol was purchased from Uvasol. Acrylamide was supplied from Bio-Rad. All other chemicals were of analytical grade and purchased from Merck with the exception of the phospholipids that were purchased from Avanti Polar Lipids. All chemicals and materials were used without further purification. Silicon substrates for template-stripping were purchased from Crystec (ultraflat substrates) and Si-Mat. DPhyTL was synthesized by Celestial Synthetics (https://www.celestialsynthetics.com).

### 2.2 Substrate Preparation

Silicon wafers were sonicated in acetone, then ethanol, and afterward ultrapure water for 10 min. The wafers were then cleaned using an acidic piranha solution containing a 3:1 mixture of sulfuric acid and a 33% hydrogen peroxide solution for 60 min. They were then rinsed thoroughly with ultrapure water and chromatography-grade ethanol and dried in a stream of nitrogen.

50 nm of 99.99% gold (MaTeck) was then deposited by thermal evaporation at a rate of 0.1 Å/s (6 A current, 10^–6^ mbar). An optical *xy*-stage with a micromanipulator was used to mechanically limit the surface area of the microelectrodes to 1000–2000 μm^2^. Polytetrafluorethylene (PTFE) tape (3 M) was used to further ensure the limitation of the microelectrode patch and avoid undesired electrical contact.

Standard microscopy slides (VWR) were then applied to the gold surface by using EPO-TEK354 optical adhesive. The glue was mixed at a 10:1 ratio of monomer/curing agent and degassed under vacuum at 50 °C for 1 h before use. Glue deposition was at 110 °C for 10 min followed by curing at 160 °C for 2 h. This procedure was adapted and further developed as originally shown by Vogel et al.^38^ The template stripping process is shown schematically in Figure S1.

### 2.3 Solvent-Assisted Monolayer Formation

The substrates were rinsed with ethanol before being inserted into an ethanolic solution of 100 μM DPhyTL and 400 μM mercaptoethanol for 18 h at 4 °C to allow the formation of the tethered monolayer.

### 2.4 Solvent-Assisted Bilayer Formation

The SAM-functionalized substrate was incubated with 100 μL of a 10 mg/mL DPhyPC solution in ethanol at 30 °C. After 10 min, the cell was flushed with 5 cell volumes of 1× PBS at a rate of 5 mL/min. Care must be taken not to exceed this rate, as otherwise the bilayer is damaged by the turbulence induced by the flushing process. However, acceptable flow rates are highly dependent on the geometry of the flow cell and should thus be optimized for each device individually.

### 2.5 Bilayer Formation by Vesicle Fusion

For the formation of OR-Orco-containing stBLMs, liposomes were prepared by extrusion (21×) through track-etched polycarbonate membranes (50 nm pores) using the Avanti mini-extruder kit. 50 μL of the liposomes was then immediately added to the substrate with a 1× PBS solution and incubated for 18 h at 30 °C. The membrane was then rinsed with 5 cell volumes of 1× PBS solution at a rate of 0.1 mL/s and monitored for stability over a period of 2 h before commencing the experiment.

### 2.6 Or22a/Orco Expression and Purification

The proteins were expressed and purified as described previously.^22^ The purified protein was inserted into preformed DPhyPC liposomes as follows. Liposomes were first made by drying chloroform-solubilized lipids under nitrogen and then in a vacuum desiccator until all solvent had been removed and then solubilizing in 10 mM HEPES pH 7.5, 300 mM NaCl to 40 mg/mL. Ten freeze/thaw steps were performed, transferring the tube from liquid nitrogen to a 40 °C water bath. Liposomes were then sized by passing the lipid solution 11× through a 100 nm polycarbonate membrane using an Avestin LiposoFAST extruder unit.

Prior to the addition of the Orco subunit, liposomes were diluted to 2 mg/mL in 10 mM HEPES, 300 mM NaCl at pH 7.5. CHAPS was added to 0.2%, and the liposomes were rotated at room temp for 15 min to destabilize. 100 μg/mL protein was added to the liposomes, and they were rotated for a further hour. 1 g/mL Bio Beads was added to the mixture and then placed at 4 °C overnight with mild agitation on a shaking platform. The liposomes were removed from the biobeads and stored at −80 °C until further use.

### 2.7 OBP Expression and Purification

The recombinant protein EcorOBP15-m1 was expressed in *E. coli* using standard protocols.^39,40^ In order to solubilize the protein, the pellet was dissolved with 8 M urea/5 mM DTT for 1 h at room temperature and then dialyzed for 3 days against 50 mM Tris HCl pH 7.4 at 4 °C. The solubilized protein was then purified by two chromatographic steps on anion-exchange HiPrep-Q (GE-Healthcare) column. All samples obtained from the expression and purification were analyzed by SDS-PAGE (Figure S2).

### 2.8 Competitive Binding of Ligands to Odorant Binding Proteins

Ligand binding experiments were performed by using a PerkinElmer FL 6500 spectrofluorometer in a right-angle configuration at room temperature and quartz cuvettes with a 1 cm path. *N*-Phenyl-1-naphthylamine (1-NPN) was used as a fluorescent probe at an excitation wavelength of 337 nm, and the emission spectra were measured from 380 to 450 nm. In order to reach concentrations of 2–16 μM, aliquots of a 1 mM methanol solution of 1-NPN were added to a 2 μM solution of the protein in 50 mM Tris–HCl buffer, pH 7.4. Intensity values were recorded at a peak maximum at 421 nm for EcorOBP15-m1. Prism software was used to calculate the dissociation constant of the complex protein/1-NPN (https://www.graphpad.com/scientific-software/prism/). The affinity of ethyl hexanoate was evaluated by adding to a mixture of the protein and 1-NPN at 2 μM concentration in 50 mM Tris–HCl buffer (pH 7.4), aliquots of 1 mM methanol solutions of the ligand to final concentration values of 2–16 μM. Dissociation constants of the ligands were calculated from the corresponding [IC]_50_ values, using the equation
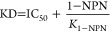
1where [IC]_50_ is the concentration of each ligand halving the initial value of fluorescence, [1 – NPN] is the concentration of free 1 – NPN, and *K*_1–NPN_ is the dissociation constant of the complex protein/(1 – NPN). The data can be found in Figure S3.

### 2.9 Electrochemical Impedance Spectroscopy

#### 2.9.1 Experimental Section

Electrochemical impedance spectroscopy (EIS) measurements were made using a Metrohm Autolab potentiostat with two FRA-modules using a three-electrode setup. The counter electrode was a Pt wire, the reference electrode was a Ag/AgCl electrode in a 3 M KCl buffer solution, and the gold substrate was the working electrode. For each spectrum, a minimum of 30 data points were collected in the range from 100 kHz to 5 mHz with an amplitude of ±10 mV. All measurements used a 1× PBS electrolyte.

#### 2.9.2 Data Analysis

**Figure 3:**
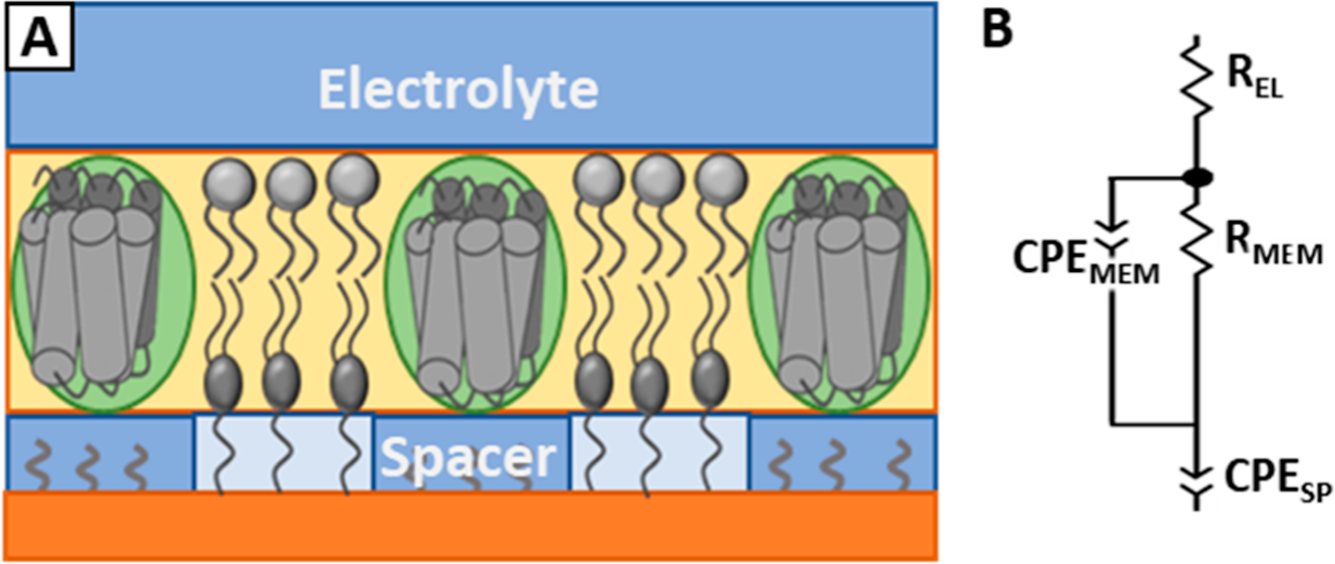
(A) Lipid bilayer approximated as matrix comprising mixed media with different dielectric properties. The olfactory receptors (green) are suspended in the lipid bilayer phase (yellow)—similarly, the submembrane region is a mixed phase system composed of anchorlipid (light blue) and water (dark blue). (B) Simplified equivalent circuit commonly used to describe lipid bilayers comprising a *R*_EL_ (electrolyte resistance), CPE_MEM_, and *R*_MEM_ describing the capacitance (as a constant phase element—CPE—which accounts for the nonideality of real systems) and resistance of the lipid bilayer, respectively, and CPE_SP_ describing the capacitance of the spacer.

EIS data were analyzed using ZView2 (Scribner Associates). Unless otherwise stated, all spectra were normalized to an electrode area of 0.28 cm^2^. The data were fitted to one of the two equivalent circuits shown in Figure S4. The error is the range of values that can be fitted for the respective parameter without decreasing the quality of the fit (evaluated by least-squares fitting). While the inclusion of CPE_SP_ was sometimes necessary to obtain a good fit for R2 and CPE_MEM_, we do not show the values of the capacitance fitted to CPE_SP_ because no useful information can be gained from it, as the associated error always exceeds the magnitude of the parameter. The large error is caused by the limited number of data points available to fit the feature (often only 1–2 data points in the low mHz range), preventing it from being fitted with any confidence. It is not feasible to record additional data points below 5 mHz as each additional data point requires 30 or more minutes to record, at which point membrane properties have often changed as ion channel opening occurs significantly faster than that. Furthermore, recording data below 5 mHz is vulnerable to interference from external electromagnetic fields, even when measuring inside a Faraday cage. The measurement setup is shown, and the equivalent circuits used to fit the EIS data are shown in Figure 3. A second resistor can be added in parallel to R2 representing the resistance of the lipid bilayer, but this has no impact on the spectrum, as no current will flow through this resistor if lower resistance pathways are available through the open receptor. Similarly, an additional CPE representing membrane capacitance can be added in parallel to CPE1, which also does not affect the impedance spectrum.

### 2.10 Single-Channel Measurements

Single-channel recordings on tethered membranes were performed using a HEKA EPC10 patch-clamp amplifier with PatchMaster software and a two-electrode setup using a AgCl-coated silver wire as a reference electrode and the gold electrode as a recording electrode. The setup was placed on a vibrationally isolated table with two grounded Faraday cages. All measurements were carried out in 1× PBS electrolyte solution. The holding potential was ±80 mV; measurements were taken for 10–12 s using a 10 kHz and a 2.9 kHz band filter as well as a 20 μs stimulus filter in two-electrode mode to remove the effect of sudden potential changes.

#### 2.10.1 Tip-Dip

Experiments were conducted using 4 in. patch clamp glass micropipettes with a 1.5 mm diameter (WPI, PG10150-4). After the pipettes were pulled to around 1 μm in diameter, they were filled with buffer solution containing vesicles and assembled into the HEKA EPC 10 patch-clamp system. A 200 μL reservoir containing a monolayer with DPhPC in trichloromethane was repeatedly penetrated with the tip until bilayer formation with giga-ohm resistance was obtained. The holding potential was ±80 mV; measurements were taken for 10–12 s using a 10 and a 2.9 kHz band filter.

### 2.11 Atomic Force Microscopy

AFM measurements were conducted using a Cypher ES (Oxford Instruments, United Kingdom) on a vibration-isolated table. Images were recorded in 1× PBS under ambient pressure and temperature. In this setup, an ARROW-UHFAuD gold-coated silicon cantilever (NanoWorld AG, Switzerland) with resonance frequencies between 2 MHz in air and 650–700 Hz in liquid was used. Operation of the AFM was conducted through blueDrive technology in tapping mode (TM-AFM) with scan rates between 2 and 8 Hz.

### 2.12 Surface Plasmon Resonance and Surface Plasmon-Enhanced Fluorescence Spectroscopy

For the spectral analysis of the bilayer formation on the gold substrate, SPR was used in the Kretschmann configuration geometry where a 2 nm chromium (Cr) and 50 nm gold (Au) (MaTeck, Germany) layer which was evaporated on a LaSF9 glass slide via thermal evaporator (HHV Ltd., Auto306 Lab Coater, UK), and high refractive index immersion oil (Cargille Laboratories, USA) was used to optically match the LaSF9 glass prism. The sample was positioned on a rotating stage that allowed for adjusting the angle of incidence. The intensity of the reflected HeNe laser beam, *R*, was measured using a photodiode after it interfered with the surface of the sensor chip at a wavelength of λ_ex_ = 632.8 nm. The fitting parameters to determine layer thickness can be found in Table S1 in the Supporting Information. Samples were all prepared in 1× PBS buffer flowed over the sensor surface using a transparent flow cell clamped to the sensor chip. A thin (300 μm) PDMS gasket that specified a flow-cell volume of 10 μL was placed between a glass substrate and a flow cell. Tygon tubing with an inner diameter of 0.25 mm was used to link the flow cell to a peristaltic pump, which was used to circulate samples that were held at room temperature at a flow rate of 40 μL/min. Surface plasmon-enhanced fluorescence measurements were carried out using NileRed dye (Thermo Fischer, λ_em_ = 635 nm/λ_ex_ = 559) to label lipid bilayers. Fluorescence was induced using a 532 nm HeNe or diode laser (Edmunds Optics, Germany), and the fluorescence signal was recorded with a photon counter (53131A from Agilent, USA) in counts per second (cps). The instrument was operated with dedicated software (Wasplas, Max Planck Institute for Polymer Research, Mainz, Germany).

## 3 Results and Discussion

### 3.1 Confirmation of Bilayer Formation by Electrochemical Impedance Spectroscopy

To characterize an ion channel, particularly at the single-channel level, it is necessary to form high-resistance lipid bilayers with minimal defects that retain their stability for periods that exceed the duration of any experiments. This ensures that changes in stBLM resistance can be confidently attributed to ion channel activity and not the formation of defects. The inner leaflet was formed on the gold substrate by overnight incubation at 4 °C in an ethanolic solution containing the anchorlipid. Bilayers were then formed by the overnight incubation of liposomes containing OR22a/Orco with the substrate.

EIS showed that upon bilayer formation, stBLM resistance increased by around an order of magnitude and its capacitance halves (see Figure S5 and Table S2). Typically, resistances were in the range of 10–50 MΩ cm^2^, which is similar to previously published DphyTL-based membranes.^25,35,41^ The capacitance of OR22a/Orco stBLMs is approximately 10 μF cm^–2^, which is significantly higher than the typical capacitance of lipid bilayers of 0.5–1 μF/cm^–2^.^32,42^ Similarly high capacitances have been reported for tethered membranes containing cytochrome c oxidase and the insect olfactory receptor coreceptor.^25,44^ The capacitance of protein-free lipid bilayers cannot be directly compared to stBLMs containing the OR22a/Orco complex as the equivalent circuit used to fit the data contains a constant phase element (CPE) instead of a capacitor to obtain good fits. This element is described with a CPE coefficient (*Q*) instead of a capacitance with an impedance (frequency-dependent resistance) of
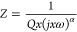
2where α = 0 for a purely resistive element and 1 for a purely capacitive element. The units of *Q* are μF cm^–2^ s^α–1^, describing capacitive behavior that includes time-dependent dipole relaxation processes. These are often found in heterogeneous dielectric materials, and we have previously reported stBLM systems containing the SthK ion channel with similar behavior.^43^ These can be found in the Supporting Information with the EIS data.

An explanation for the increased capacitance of heterogeneous sparsely tethered membrane systems was proposed by Jeuken and colleagues,^45^ who observed interfacial polarization effects in an stBLM comprising EO3C, a cholesterol-based (anchorlipid and mercaptohexanol) that caused frequency-dependent capacitive behavior known as Maxwell–Wager–Sillar (MWS) polarization caused by dielectric dispersion (a distribution of dipole relaxation times) in heterogeneous films. This is an appropriate description of stBLMs assembled on mixed SAMs due to the occurrence of microdomain formation where patches of spacer and lipid are formed.^45^ MWS or effective medium models are frequently used to describe the behavior of films comprising a matrix (phase 1) in which a second phase is suspended in the form of microspheres, which can also be used to describe a protein-containing stBLM (see Figure 3A).^46,47^ This results in a significantly increased apparent capacitance at low frequencies. For example, the aforementioned paper reports that the capacitance of a mixed SAM comprising 30% tether and 70% spacer is approximately 2 μF/cm^2^ at 1 kHz and 8 μF/cm^2^ at 100 mHz. A similar but less pronounced increase in capacitance was also observed in the capacitance of the lipid bilayers assembled on these SAMs. For example, stBLMs composed of EggPC assembled on 67% tether had an imaginary (frequency-dependent) capacitance of 3.5 μF/cm^2^. Dielectric dispersion was observed in the cell membrane of frog skin^48^ and apical skin cells,^49^ where relaxation constants in the range of applied frequencies resulted in unexpectedly high capacitances obtained via impedance measurements.

A capacitance of 2–3 μF/cm^2^ corresponds to a lipid membrane with a capacitance of 1 μF/cm^2^ containing 2–5% of an ion channel with a dielectric permittivity of approximately 60. This is reasonable given that MD simulations have shown that the relative permittivity ε_r_ can be as high as 60 inside an ion channel^50^ and that depending on the amino acid composition, the permittivity inside the protein could exceed that of water, as the permittivity of water could differ significantly from its bulk properties.^51,52^ To fully understand the phenomena causing the increase in membrane capacitance at low frequencies of protein-containing tethered membranes, a more complex model containing several capacitors and CPEs connected in parallel would likely result in a better fit, but adding parameters to the model always carries the risk of overfitting. Model development should thus be accompanied by extensive experimental work and MD simulations.

To characterize ion channels, we are primarily concerned with the membrane resistance; therefore, we use the simple electrical circuit typically employed to fit EIS data of tethered membranes (Figure 3B). To obtain the real (frequency-independent) membrane capacitance, the capacitance was calculated based on charging at a constant voltage, obtaining capacitances of 2.8 ± 0.6 and 26.2 ± 5.8 μF/cm^2^ before and after ligand addition, respectively. The data and calculations can be found in the Supporting Information (Figure S6). The large increase in capacitance can be attributed to a combination of factors. First, the membrane becomes “leaky” upon ion channel opening, and as such, the capacitance of the submembrane reservoir and the membrane itself become indistinguishable as ions accumulate in the submembrane region. Furthermore, as charged residues inside the ion channel become exposed to the aqueous environment, they also contribute to the capacitance. As discussed earlier, the details of the physical phenomena that take place will require careful combination of MD simulations and experimental work to unravel. We therefore primarily focus on the changes in electrical resistances, as they are more straightforward to interpret.

As we have shown previously, stBLMs containing only DPhyPC cannot form at 20% tethering density.^25^ However, we have found that the addition of 20–40 mol % cholesterol allows the formation of high-resistance protein-free stBLMs on this membrane architecture. We therefore used stBLMs containing 20 mol % cholesterol for control experiments with sparsely tethered membranes and repeated the experiments with inner leaflets comprising 100% DPhyTL such that the outer leaflet could be prepared with pure DPhyPC. While no cholesterol is present in OR-containing stBLMs and it is not necessary for ion channel function, insect cell membranes do contain cholesterol^53^ and therefore a cholesterol-containing receptor-free membrane is reasonable to use as control.

Stable, electrically insulating tethered membranes without protein content have been reported with a lifespan exceeding 9 months.^42^ It is unlikely that the function of embedded proteins can be maintained for more than a few days, however, and our data show excellent electrical stability with no loss of membrane resistance after 24 h and only around 50% reduction over the following 4 days (see Figure S5 and Table S2). This is sufficient to measure receptor functionality, as ion channel opening occurs in less than 1 h in this system.

### 3.2 Confirmation of Bilayer Formation by Surface Plasmon Resonance

To confirm the optical properties and thickness of the OR-Orco stBLM, we monitored the vesicle fusion process by using SPR. SPR showed the formation of a 6 nm thick layer with a refractive index of 1.48 upon stBLM formation, which is in good agreement with the reported refractive index of lipid bilayers.^54^ Film thickness reached 6 nm after 30 min and then remained stable (see Figure S7A), which is in good agreement with previous reports.^25^ To determine whether the addition of ethyl hexanoate causes the formation of water-filled defects, we incorporated the polarity-sensitive dye nile red into the stBLM. Nile red fluoresces strongly in nonpolar environments such as organic solvents or lipid bilayers but is quenched in aqueous media.^55^ No reduction in fluorescence occurred upon the addition of 100 μM ethyl hexanoate (see Figure S7B), suggesting that the addition of the ligand did not cause the formation of conductive defects that might be mistaken for ion channel activity.

### 3.3 Confirmation of Bilayer Formation and Topology by AFM

**Figure 4:**
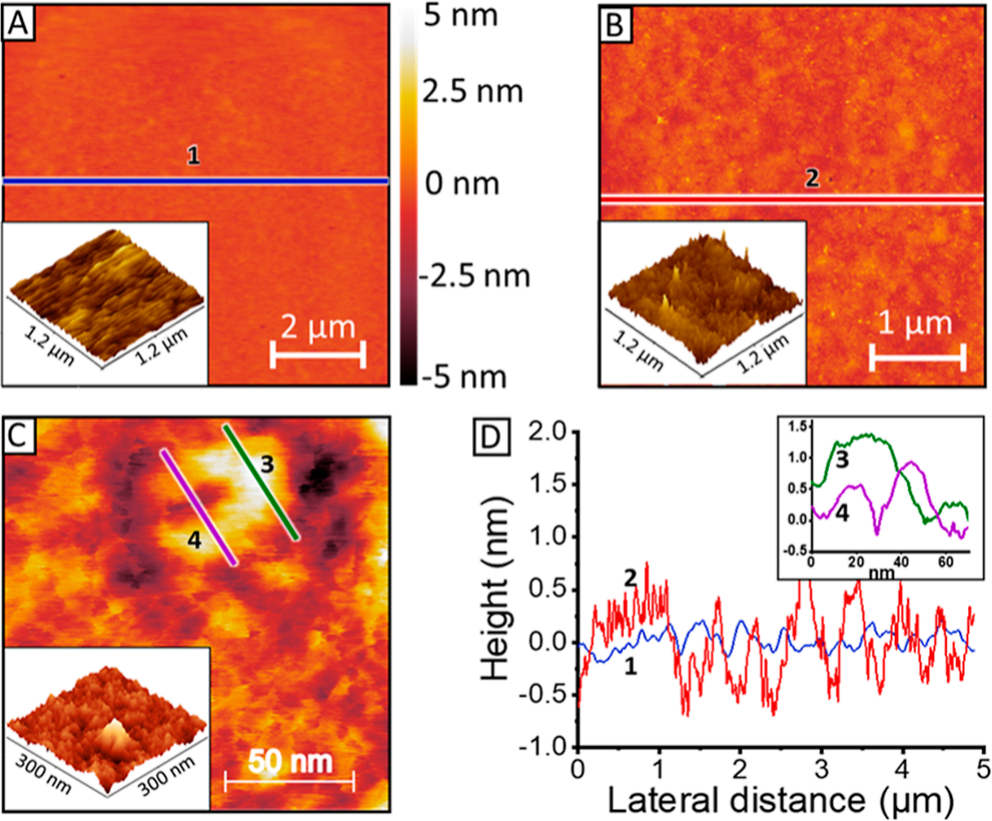
(A) AFM images of a protein-free stBLM comprising DPhyPC and 10% cholesterol, (B) OR–Orco containing stBLM, and (C) high-resolution image of the features seen in (B). (D) Representative height traces of each at the marked positions; insets of the AFM images showing 3D-rejections of the AFM data. Additional AFM data are shown in Figure S12.

To interpret EIS data, assumptions must be made about the structure of the sample, as the measured data can arise from a number of different structures on the surface, such as adsorbed vesicles and lipid multilayers. The main difference expected before and after stBLM formation is a significant reduction in surface roughness, as shown previously.^25^ Prior to bilayer formation, the substrate has an rms roughness of approximately 1.6 nm.^25^ Protein-free stBLMs (see Figure 4A) have a typical roughness of 0.2 ± 0.08 nm (*n* = 11), whereas the membrane containing OR-Orco has a roughness of 0.4 ± 0.05 nm (*n* = 11). The small increase in roughness compared with protein-free membranes can be attributed to the presence of OR-Orco. As shown in Figure 4B, OR22a/Orco-containing stBLMs show features of ∼1 nm in height and 50 nm in width. While Orco does not extend significantly above the membrane,^16^ recent modeling suggests that ORs may extend ∼1 nm above the membrane surface,^56^ which can also be seen in the AFM data shown in Figure 4. The features generally became less apparent when the resolution of the AFM image was increased, which could be due to deformations induced by the AFM tip. A higher resolution might be achieved in future studies with softer AFM tips, which have been used to resolve structures of membrane proteins showing single amino acid residues.^30^ Filtering the AFM data for features with a height of 0.8–1.2 nm yields an estimated protein density of 12.7 ± 4.3 μm^–2^ (see Figure S10 for the analysis). The receptor appears to insert into the stBLM predominantly in the correct orientation with the submembrane domain of the protein residing underneath the membrane; only very few 4 nm peaks could be seen in AFM that indicate an upside-down orientation.

Small numbers of pinhole defects with depths of approximately 5–7 nm were evident in some areas of the bilayers (see Supporting Information, Figure S12), which corresponds to the approximate thickness of a lipid bilayer (2 nm) with an addition of 3–5 nm of space between the lipid bilayer and support, affording sufficient space to accommodate the submembrane domain of Orco.

### 3.4 Confirmation of Receptor Function by EIS

The function of the incorporated ORs was confirmed by the addition of ethyl hexanoate, one of the main ligands for OR22a/Orco.^57^ As a control, we used methyl salicylate which should elicit no response in Or22a/Orco.^13,22,58^ The ion channel opening is visible in EIS as a decrease in the electrical resistance of the stBLM. However, as a decrease in electrical resistance alone is also caused by the formation of conductive defects not related to ion channel opening, electrical resistance should increase significantly upon removal of the ligands. Upon addition of 1 μM ethyl hexanoate, the measured resistance decreased by 2 orders of magnitude from 50 to 0.6 MΩ cm^2^ after an incubation time of 30 min, whereas no significant reduction in membrane resistance was measured after incubation with 1 μM methyl salicylate for 1 h. We note that 30 min is significantly longer than one would expect for the response of ion channels; we chose this waiting time to ensure that all receptors would open, as EIS measures receptor response only at the macroscopic scale. Ion channel activity could be measured immediately upon ligand addition when making single-channel recordings (see Figure 7).

**Figure 5:**
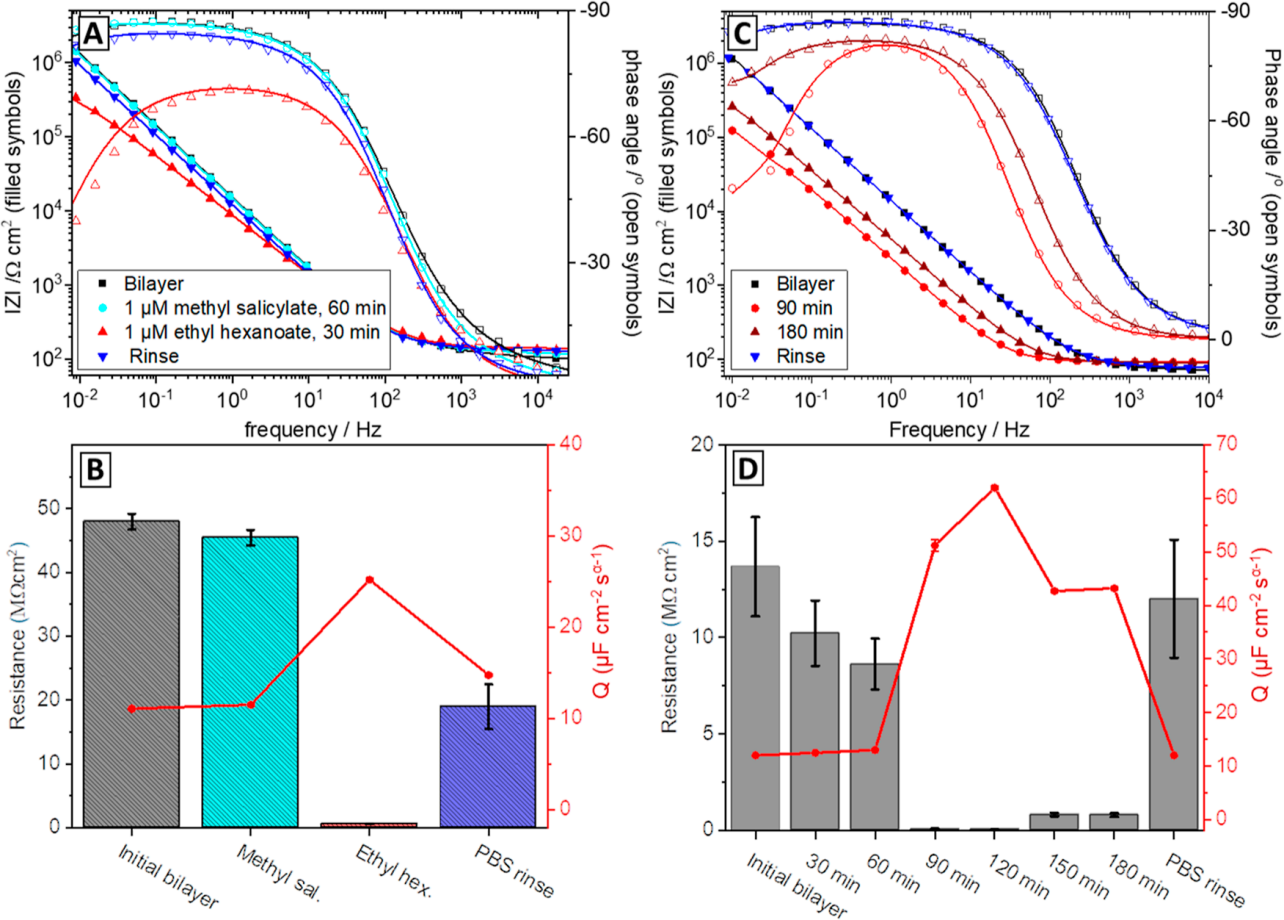
(A) EIS data showing membrane resistance upon addition of methyl salicylate and ethyl hexanoate. Experimental data are shown as symbols; fits are shown as solid lines. Electrical properties extracted from fitting EIS data are shown in (B). (C) Incubation of OR–Orco stBLM with 1 μM ethyl hexanoate over an extended period of time. Electrical properties extracted from fitting the data are shown in (D). The error bars represent the errors of the fits, indicating the range of values that can be fitted without decreasing the quality of the fit. Additional replicates of this experiment can be found in the Supporting Information in Figure S14 and Table S5. The data shown graphically in (B,D) can be found in Table S5.

**Figure 6:**
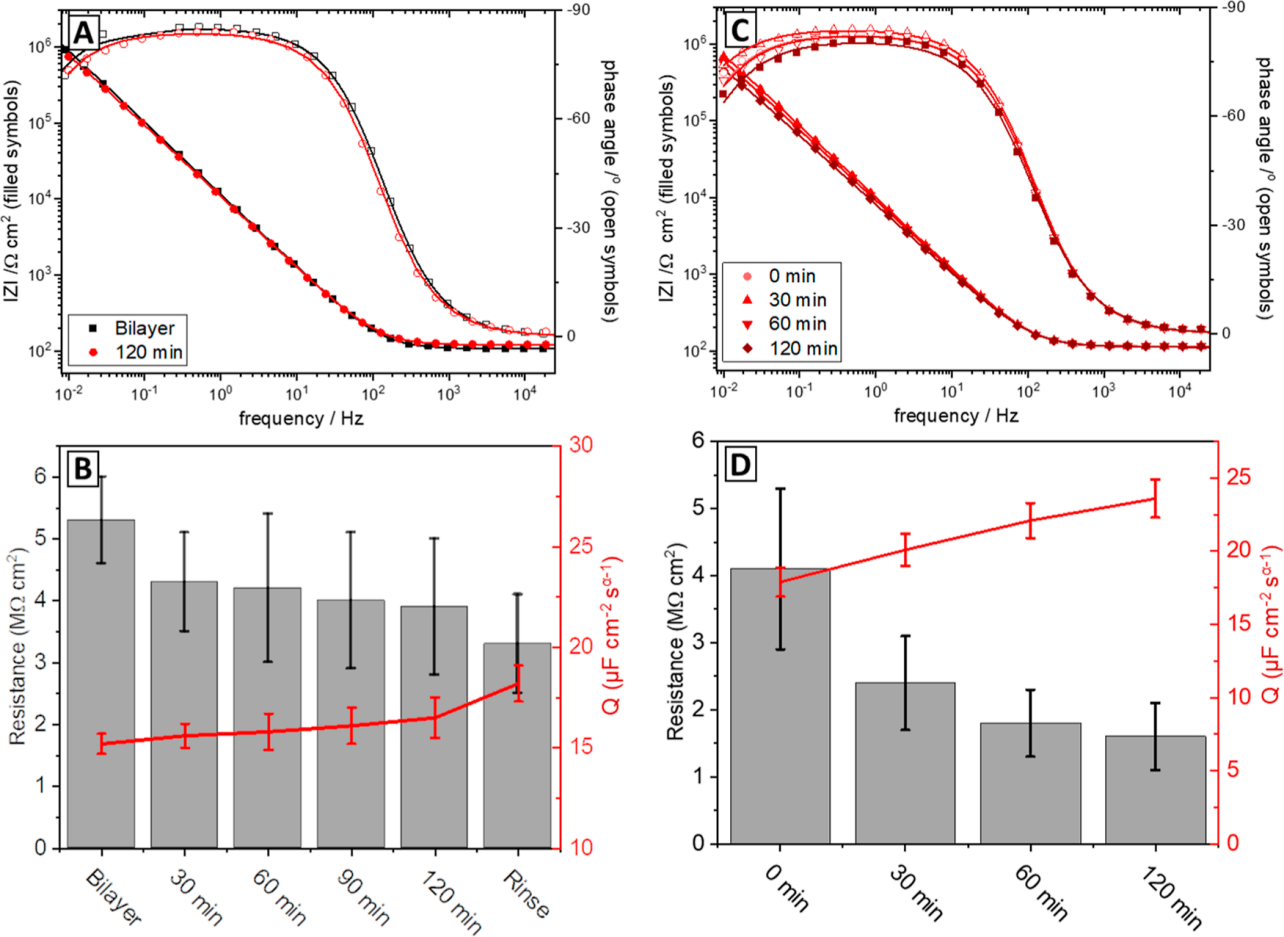
EIS data of OR–Orco stBLM where receptor activity was inhibited by tryptamine. (A) Incubation of bilayer with 1 μM ethyl hexanoate in the presence of 1 μM tryptamine. Electrical properties extracted from fitting the data are shown in (B). (C) Incubation of OR–Orco stBLM with 1 μM ethyl hexanoate after the removal of tryptamine. Electrical properties extracted from fitting the data are shown in (D). The error bars represent the error of the fits, indicating the range of values that can be fitted without decreasing the quality of the fit.

**Figure 7:**
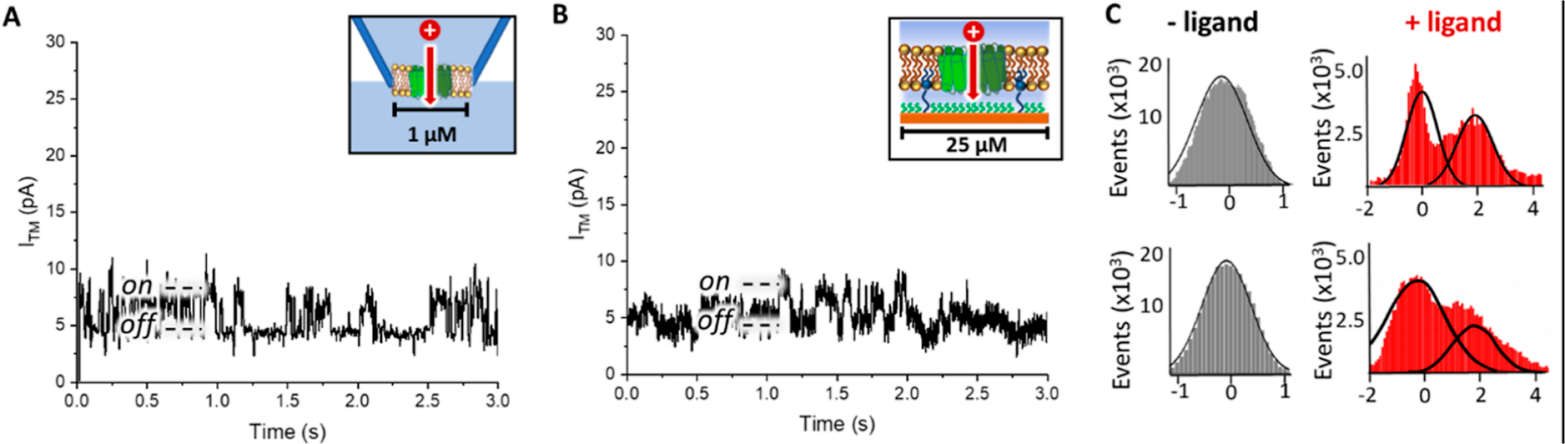
(A) Single-channel currents of OR22a/Orco recorded at 80 mV in free-standing lipid membranes prepared by the tip dip method. (B) Currents recorded in tethered membranes on microelectrodes. (C) All-point current (relative the baseline) histograms of the recordings shown in (A,B). Measurements of OR22a/Orco in tip dip membranes taken before ligand addition and additional recordings after ligand addition can be found in Figure S18. Blank recordings of the OR22a/Orco complex in tethered membranes can be found in Figure 8A.

Upon rinsing, the resistance recovered to 20 MΩ cm^2^, close to the original resistance prior to ligand addition. In some cases, full recovery of the resistance was not possible, which could indicate incomplete ligand removal, as ethyl hexanoate is poorly soluble in aqueous solvents. A longer delay between rinsing and measuring appears to eliminate this effect, as can be seen by the full recovery of the membrane resistance in the data shown in Figure 5D. As the electrical resistance of the stBLM can be compromised even by a very small number of defects that might not be visible in optical measurements, we also tested whether ethyl hexanoate affects tethered membranes comprising only DPhyPC. We found only very minor changes in membrane resistance after incubating the bilayer for two h with 8 mM ethyl hexanoate (see Figure S13 and Table S4), a concentration which is several orders of magnitude higher than what was used for experiments with OR–Orco stBLMs.

To further investigate the response kinetics of OR22a/Orco in stBLMs, we measured membrane resistance in shorter intervals after ligand addition, revealing a maximum reduction in resistance after 90 min by 2 orders of magnitude from 13 to 0.06 MΩ cm^2^ (see Figure 5C). After this point, resistance increased slowly, likely caused by a decrease in ligand concentration as it evaporated from solution due to its poor water solubility. Upon ion channel opening, the capacitance approximately doubled from 11 to 25 μF/cm^2^ compared to that in the closed state. There are likely several main contributing factors to this effect: a change in confinement in the water inside the pore,, and a potential increase in unconfined water in the pore as it changes conformation to allow charge transport and finally the presence of cations inside the membranes during the charge transport process. Due to the significant reduction in lipid bilayer resistance, the gold interface may also contribute significantly to the measured capacitance. The increase in membrane capacitance is fully reversible, however, returning to its previous level upon ligand removal. After ion channel closing, ligand addition can be repeated on the same stBLM (data shown in Figure S14 and Table S5). However, repeated ligand addition results in reduced ion channel opening, with membrane resistance only reducing by half an order of magnitude. We attribute this to the accumulation of cations in the submembrane reservoir, an effect we have observed previously in sparsely tethered membrane architectures.^36^ This can be reversed by incubating the membrane system in salt-free conditions for an extended period of time, but as the absence of a buffer might harm the protein, we did not explore this option here.

Assuming that all embedded ORs are saturated with ligands after incubation with 1 μM ethyl hexanoate for 90 min, we can estimate the number of ion channels based on the resulting stBLM resistance. Previous characterization of the conductive receptor subunit Orco has shown a conductivity of 31 pS per pore (a normalized resistance of 0.0025 Ω cm^2^). Based on the resistances of 58–140 kΩ cm^2^ after ethyl hexanoate addition, we obtain a receptor density of 2.3–5.6 μm^–2^ (see Supporting Information for full calculation). This is below the density of ∼12/μm^2^ indicated by the AFM measurements, which suggests that not all incorporated receptors were functional, although previous publications have also reported reduced conductivity of ion channels in the tethered membrane due to the restricted volume of the submembrane reservoir.^59^ Some of the differences can also be attributed to variation in nanoscale topology of the substrate, as the same sample cannot be used for EIS and characterized in AFM.

Finally, we determined whether receptor response could be inhibited by tryptamine, a compound produced by plants which is known to inhibit the olfactory response of insects.^60^ In the presence of 1 μM tryptamine, there was no significant receptor response to ethyl hexanoate over two h (see Figure 6A). The apparent drift in membrane resistance is likely an artifact of the fitting process, as the experimental data in Figure 6a do not change. Upon removal of tryptamine, a small reduction in resistance of approximately 50% could be seen after 60 min, showing that receptor inhibition was at least partially reversible (see Figure 6B).

Given the reliance on self-assembly and the nanoscale topology of the substrate to provide cavities to accommodate the receptor, there was some variation in the resistance of the stBLMs. However, our experiments show excellent qualitative reproducibility, with resistances reliably decreasing by 2 orders of magnitude upon ligand addition. Future improvements of this technology, for example, including sample preparation in a clean room environment, should significantly improve the quantitative reproducibility of the data.

### 3.5 Single-Channel Measurements in Tethered Membranes

It has been shown previously that measuring the activity of single ion channels in DPhyTL-based tethered membranes is possible. For example, Andersson et al. reported the detection of single-channel activity of Gramicidin,^59^ and Keizer and colleagues reported single-channel activity of the pore segment of the acetylcholine receptor.^61^ However, both membrane pores are relatively small and simple proteins with no submembrane domains and function in tethered membranes formed on ultraflat gold with inner leaflets comprising 100% DPhyTL. As we have shown previously, these membrane architectures are unsuitable to large, multimeric proteins with significant submembrane domains.^25^

The Orco pore has a reported conductivity of 20–30 pS per ion channel, resulting in currents of ∼2.5 pA at 80 mV.^16,25^ To study receptor function at the single-channel level, the noise level must therefore be reduced below ∼1 pA. At an applied potential of 80 mV, stBLM resistance should therefore reach at least 10 GΩ, with higher resistances being desirable as they further reduce background current, allowing the use of increased amplifier voltage. To predict the expected direction of the peaks of the single-channel currents, the actual transmembrane potential must be determined. The potential of zero free charge (PZFC) in sparsely tethered membranes has been reported to be approximately 0.3 V.^62^ From this, the transmembrane potential can be determined using the following equation
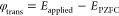
3

**Figure 8:**
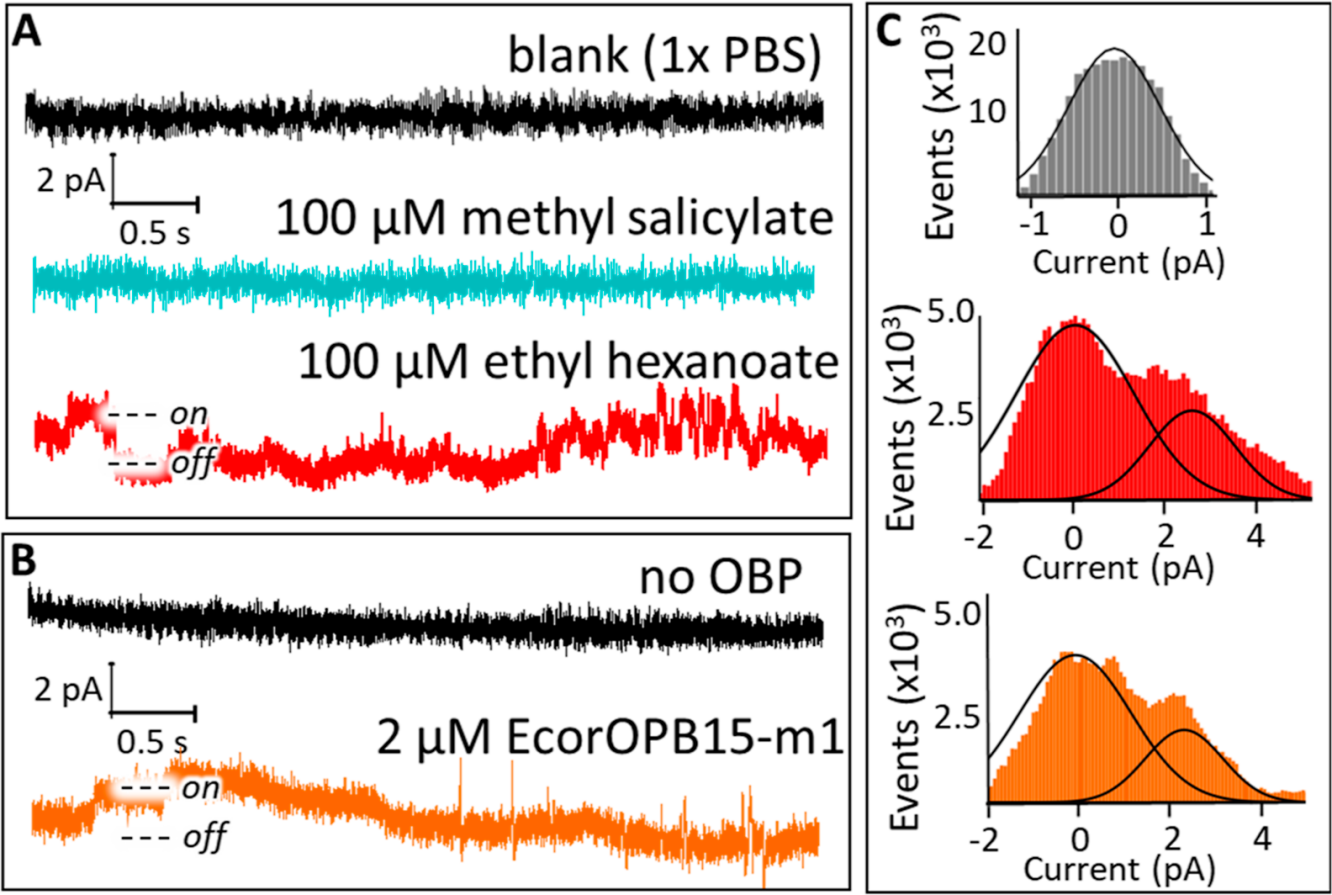
(A) Single-channel measurements before the addition of ethyl hexanoate (black), after addition of 100 μM methyl salicylate (cyan), and after addition of 100 μM ethyl hexanoate (red). (B) Receptor activity triggered by gaseous ethyl hexanoate bubbled through PBS solution (black) and PBS containing 2 μM EcorOBP 15 mL (orange). The second step in the current trace is caused by the opening of an additional ion channel in the membrane patch being measured. (C) All-point current histogram of receptor response. Black lines show deconvolution of the data into one (top) or two separate peaks (middle and bottom). Transmembrane potential was set to 80 mV. The data shown here is the raw experimental data without any additional signal processing or noise reduction beyond the filter applied by the patch clamp amplifier.

This results in a PZFC of ∼−220 mV if a potential of 80 mV is applied at the working electrode for single-channel measurements. The ion channel conducts cations through the lipid bilayer into the submembrane reservoir along this potential gradient, resulting in positive single-channel current peaks, which is what can be seen in the single-channel recordings in Figures 8 and S20–S22.

### 3.6 Electrode Design

We achieved suitably high resistance by decreasing the area of the lipid bilayers to ∼1000 μm^2^. Images of the electrodes can be found in the Supporting Information in Figure S15. stBLMs formed on these electrodes reached resistances of 40–50 GΩ or more (see Figure S16 and Table S6), resulting in noise levels of 1 pA or below (see Figure 8). Chemically tethering the lipid bilayer to the support significantly increased the robustness of the membrane system, which easily tolerates mechanical disturbances caused by moving the cell around without the need for particular care and allowed us to use a simple measurement cell (Figure S17). Overall, this represents a significant improvement in useability compared to traditional patch clamping experiments, eliminates the need for an optical microscope, and reduces the training required to perform experiments, as stBLMs are formed via self-assembly. The success rate of measuring single-channel activity in stBLMs was approximately 50%. As each substrate is manufactured with 4 microelectrodes, this leads to an average of two successful experiments per substrate. Failure was caused primarily by electrode defects, mostly due to substrate damage or short circuiting. Improvements in the manufacturing process should therefore eliminate the majority of experimental failures.

### 3.7 Measurement of Single-Channel Activity

While the conductivity of the Orco-tetramer by itself has been published,^16^ there are no literature values on the single-channel behavior of the OR22a/Orco complex. It is likely that the single-channel currents of the OR22a/Orco complex are similar to those of the Orco pore, with a spike amplitude of approximately 2 pA. However, as Orco can be triggered only by the synthetic ligand VUAA1, the response of the OR22a/Orco complex to naturally occurring ligands may differ. To observe the conductivity of OR22a/Orco in a more established reference system, we incorporated it into free-standing lipid bilayers prepared via the tip dip method, observing spikes with amplitudes of approximately 2–3 pA (see Figure 7A) upon addition of ethyl hexanoate. In a tethered membrane system, we recorded a very similar activity upon ligand addition (see Figure 7B). The success rate of recording single-channel activity of OR22a/Orco in free-standing membranes prepared via the tip dip method was approximately 5% (ca. ten times lower than in stBLMs), as bilayers often rupture before recordings can be made.

Figure 8A shows spikes with amplitudes of ∼2.5 pA upon addition of 100 μM ethyl hexanoate, which are characteristic for single-channel activity, and no response is seen upon addition of methyl salicylate. We used concentrations in single-channel experiments than for the EIS experiments described earlier to ensure reliable receptor response, as ethyl hexanoate rapidly evaporates from solution due to its poor solubility. Additional single-channel recordings upon ethyl hexanoate addition can be found in Figure S19. Rinsing of the stBLM with PBS buffer eliminated channel activity (Figure S20)

Control experiments showing ligand addition to protein-free stBLM system control can be found in the Supporting Information in Figure S21. As an additional control, we repeated the addition of ethyl hexanoate to substrate functionalized only with a mixed SAM comprising 20% DPhyTL and 80% mercaptoethanol. At 140–170 pA, background currents through the SAM were significantly higher than through the lipid bilayers, and no response could be seen upon the addition of ethyl hexanoate (see Figure S22). For all experiments, a decrease in channel activity over time was observed, likely caused by the evaporation of the ligand from solution as observed in the EIS experiments. Repeated experiments on the same membrane patch resulted in reduced receptor activity, as the buildup of charge underneath the membrane eventually reduces the transmembrane potential to zero and no further ions can flow, and the built-up charge requires 5–10 min to fully dissipate. The charge transport process differs somewhat between free-standing lipid bilayers and tethered membranes, as free-standing membranes offer a quasi-infinite reservoir for ions, whereas tethered membranes have a restricted reservoir below the membrane.

However, the change in cation concentration in the submembrane reservoir due to single-channel activity is relatively minor. We can calculate the change in electrolyte concentration in the submembrane reservoir based on the current of the single-channel conductivity. One pA corresponds to a charge transfer for 10^–12^ C/s, and as a single cation possesses a charge of 1.6 × 10^–19^ C, this is a total charge transfer of 6.25 × 10^6^ ions/s or 10^–17^ mol/s, which is in a similar range as the single-channel currents reported for Gramicidin in tBLMs.^59^ Based on the depth of the defects shown by AFM (see Figure S12), a reasonable estimate of the average height of the submembrane space is 4 nm. This results in a total submembrane volume of 4 × 10^–15^ L on a 1000 μm^2^ electrode and a concentration change at a current of 2 pA of approximately 5 mM/s. The transport of sodium ions into the submembrane reservoir depletes the ions accumulated on the outside of the membrane. However, at a transmembrane potential of −220 mV, the lipid bilayer accumulates approximately 1.4 × 10^10^ sodium ions (assuming a bilayer capacitance of 10 μF/cm^2^ and an electrode area of 10^4^ μm^2^). During the maximum measurement period of 10 s, an uninterrupted 2 pA current across the lipid bilayer would consume 1.3 × 10^8^ ions, less than 1% of the total accumulated charge. The limiting factor, as discussed earlier, is the period of time required for dissipation of the accumulated charge in the submembrane reservoir.

Statistical evaluation of the single-channel recordings (Figure 8C) shows that the data are composed of a large peak with a normal distribution around 0 pA representing the noise caused by background current leakage. An additional peak at 2–2.5 pA appears upon ligand addition, which is approximately 0.5 pA higher than the currents that were reported by the activation of Orco by VUAA1.^16^ Considering that these measurements were made using a structurally different receptor and a different ligand, this deviation is not surprising.

### 3.8 Detection of Airborne Ethyl Hexanoate

**Figure 9:**
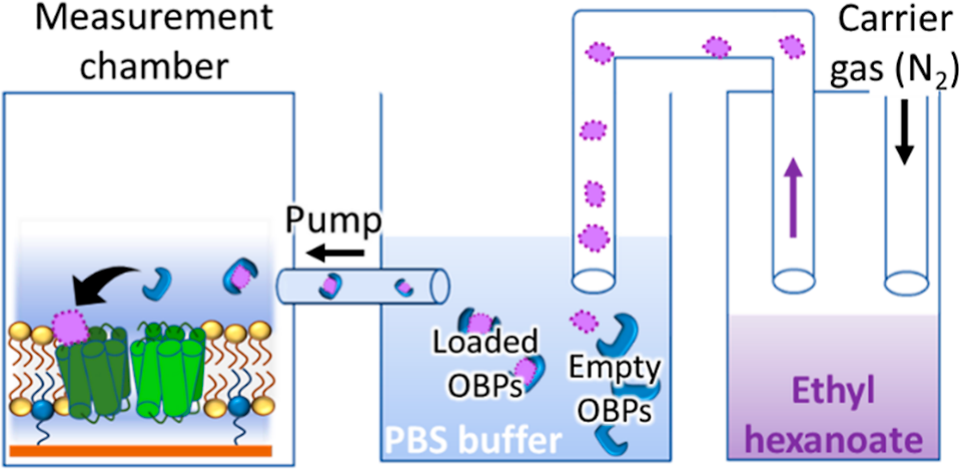
Setup used to measure single-channel activity induced by airborne ethyl hexanoate.

To fully replicate the olfaction process, receptor activity should be triggered by airborne odorants rather than the addition in an aqueous solution. To collect samples from air, a stream of nitrogen containing ethyl hexanoate was passed through the buffer solution before it was passed over the stBLM (see Figure 9 for a schematic of the experimental setup). We chose this design to separate the nitrogen stream from the single-channel measurements, as it would have likely added significant additional noise to the measurements and reduced reliability. When no OBP was present in the buffer solution through which the gas was passed, there was no channel response (Figure 8B, black). Next, we tested whether the OBPs could capture the ligand from the gas passed through the buffer solution.

As the ligand affinities for *D. melanogaster* have not yet been studied, we could not use OBPs from this organism. Instead, we screened the OBPs available in our laboratory to determine whether any of them were able to bind ethyl hexanoate and found EcorOBP 15 mL, a derivative of EcorOBP15 from *Eupeodes corollae,* in which Phe61 was replaced by Leu. EcorOBP 15 mL has a *K*_D_ of 7.4 μM with ethyl hexanoate (see Figure S3). To determine whether the OBP was able to transfer ethyl hexanoate to the olfactory receptor, we incubated a 2 μM solution of the OBP with ethyl hexanoate from 1 to 100 nM to test whether the presence of the OBP increased receptor sensitivity. When added in combination with the OBP, the receptor response could be measured down to a concentration of 10 nM (see Figure S16 and Table S6). This may indicate that the sensitivity of the receptor is increased when ligands are delivered via an odorant binding protein , but it could also be a result of the reduced evaporation of ethyl hexanoate from solution if it is bound to an OBP.

When the gas carrying ethyl hexanoate was passed through a buffer solution containing 15 mL of 2 μM OBP, ion channel activity could be observed (Figure 8B, orange). Additional data showing single-channel activity triggered by airborne ethyl hexanoate are shown in Figure S23. While the interaction between OBPs and ORs remains poorly understood,^63^ these data clearly show the role of OBPs not only in binding airborne ligands but also in transporting them to the OR and eliciting the receptor response.

## 4 Conclusions

We have presented the functional incorporation of the insect Orco complex OR22a/Orco into a stable, sparsely tethered membrane system. The architecture is prepared exclusively via self-assembly, stable at room temperature for several days, and has a high tolerance for mechanical disturbances. The stBLM reached resistances of 20–50 GΩ, allowing the recording of single-channel activity. Using EIS, optical methods, and AFM, we have thoroughly investigated the structure and function of the stBLM, demonstrating the wide range of analytical tools that can be applied to tethered membrane systems. Using this sparsely tethered lipid bilayer nanoarchitecture, we reproduced the process by which OBPs capture hydrophobic airborne ligands and transport them across aqueous reservoirs to the OR–Orco complex. In addition to allowing real-time, label-free observation of olfactory signaling, the ability to detect single-channel activity triggered by airborne compounds can serve as highly sensitive platforms for biosensors that reproduce the exquisite sensitivity of insect olfaction, which will be of enormous benefit for applications in biosecurity and agriculture. Model membranes also have the potential to greatly improve our understanding of the dielectric behavior of membrane proteins, particularly in ion channels. To fully realize this potential, an understanding of the structure of these systems at the molecular level is necessary. These can be obtained by a combination of molecular dynamics simulations and neutron scattering, as was done by Hoogerheide and colleagues recently with a tethered membrane system containing a voltage-dependent anion channel.^64^ The model system we present here offers a unique opportunity to study ion channel behavior predicted by simulations (see, for example, the work of Shrivastava and colleagues and Park et al.),^65^ as they offer a controlled and customizable experimental environment to test predictions made in silico.^66^

## Supporting Information Available

Additional replicates of experiments, extended description of the optical characterization of the membrane system, and further experimental methods including photographs of the experimental setup (PDF)
