# Supplementary material for: Activity of Single Insect Olfactory Receptors Triggered by Airborne Compounds Recorded in Self-Assembled Tethered Lipid Bilayer Nanoarchitectures
Source: ACS Appl Mater Interfaces. 2023 Sep 27;15(40):46655–67. doi: 10.1021/acsami.3c09304 (PMC10571041; doi:10.1021/acsami.3c09304)
Supplement: Supplementary file 1 — am3c09304_si_001.pdf [file am3c09304_si_001.pdf]

SUPPORTING INFORMATION

Activity of single insect olfactory receptors triggered by airborne compounds recorded in self-assembled tethered lipid bilayer nanoarchitectures

David Kleinheinz[1], Chiara D'Onofrio[1], Colm Carraher[2], Anil Bozdogan[1], Ulrich Ramach[3,4], Bernhard Schuster[5], Manuela Geiß[6], Markus Valtiner[3], Wolfgang Knoll[1,7] and Jakob Andersson* [1, 3]

[1] Austrian Institute of Technology GmbH, Giefinggasse 4, 1210 Vienna, Austria

[2] The New Zealand Institute for Plant and Food Research, 120 Mount Albert Road, Sandringham 1025, Auckland, New Zealand

[3] Technische Universität Wien, Wiedner Hauptstr. 8-10/134, 1040 Wien, Austria

[4] CEST Kompetenzzentrum für Oberflächentechnologie, Viktor Kaplan-Straße 2, 2700 Wiener Neustadt, Austria

[5] University of Natural Resources and Life Sciences (BOKU), Department of Bionanosciences, Institute of Synthetic Bioarchitectures, Muthgasse 11, 1190 Vienna, Austria

[6] Software Competence Center Hagenberg GmbH, Softwarepark 32a, 4232 Hagenberg, Austria

[7] Danube Private University, Steiner Landstraße 124, 3500 Krems an der Donau, Austria

* Correspondence should be addressed to: jakob.andersson@ista.ac.at

Substrate preparation by template-stripping

Surfaces with precisely controlled surface characteristics can be obtained by the process shown below in . The deposited gold is covered with a resin covered glass slide, once thermally cured, will allow a stripping between the Si-Au layer. The newly obtained gold interface is then exposed to an ethanolic solution, forming a monolayer on the gold surface. The silicon substrate is removed immediately prior to insertion into the solution to form the SAM, rinsed thoroughly with ultrapure ethanol and dried in a stream of nitrogen.

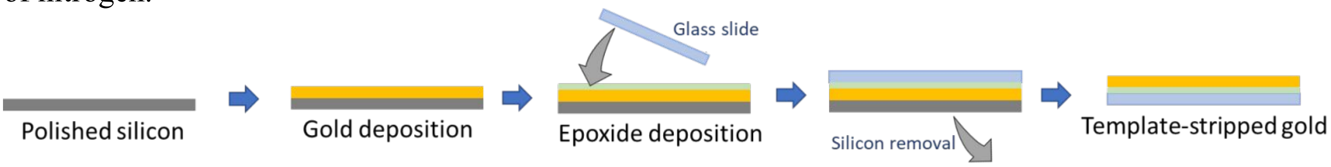

Figure S 1: Overview of the template stripping process.

OBP expression

All the samples obtained from the expression and purification were analyzed by SDS-PAGE (Figure S1).

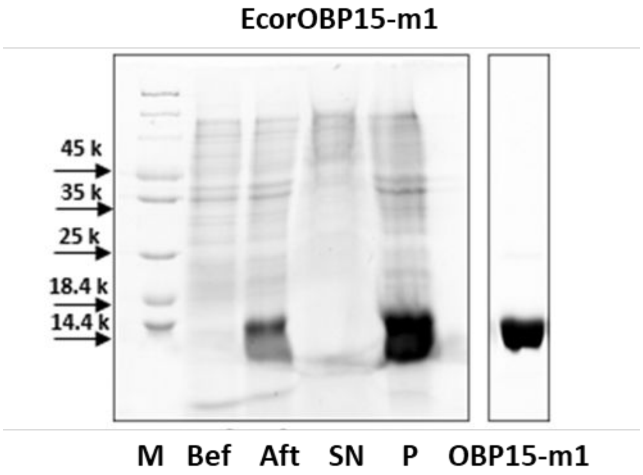

Figure S 2: Bacterial expression and purification of EcorOBP15-m1. M indicates the molecular weight markers; Bef and Aft are the crude extract collected before and after inducing the expression with IPTG; SN, P and OBP15-m1 refer to the supernatant, the pellet and the pure protein obtained after two steps of anion exchange chromatography, respectively.

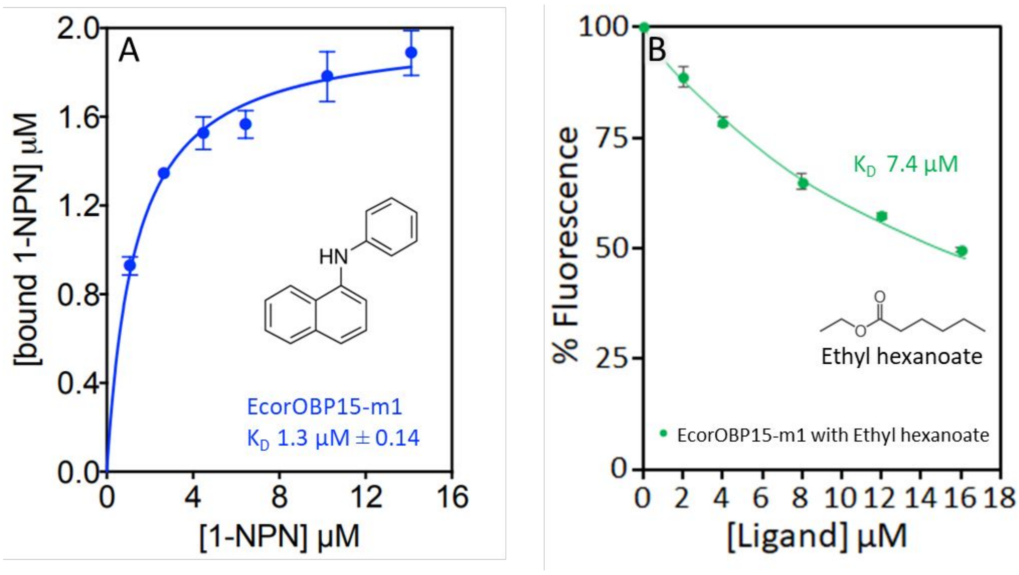

Figure S 3: A) NPN (N-phenyl-1-naphtylamine) binding to EcorOBP15-m1. B) Determination of EcorOBP15-m1 affinity for ethyl hexanoate by fluorescence displacement. The data shown are the average of three replicates of the binding assay, with error bars showing the standard deviation.

Confirmation of bilayer formation by EIS

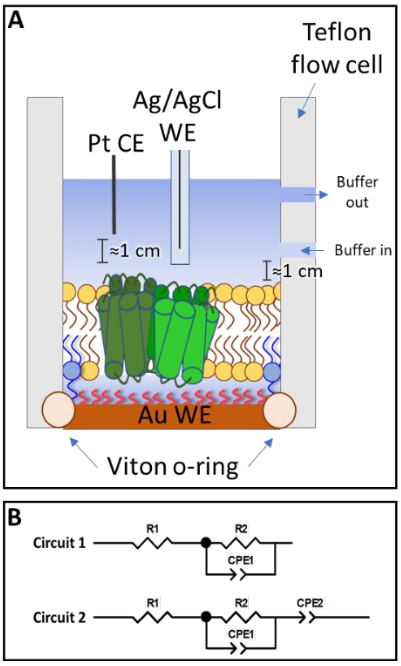

Figure S 4:A) Configuration of the measurement cell for EIS and single channel measurements. B) Equivalent circuits used to fit the EIS data. R1 represents the electrolyte. R2 and CPE1 represent the resistance and capacitance of the lipid bilayer or, when the ion channels have opened, the resistance & capacitance of the ion channels. CPE2 represents the Helmholtz double layer capacitance of the interface between the sub-membrane space and the gold electrode.

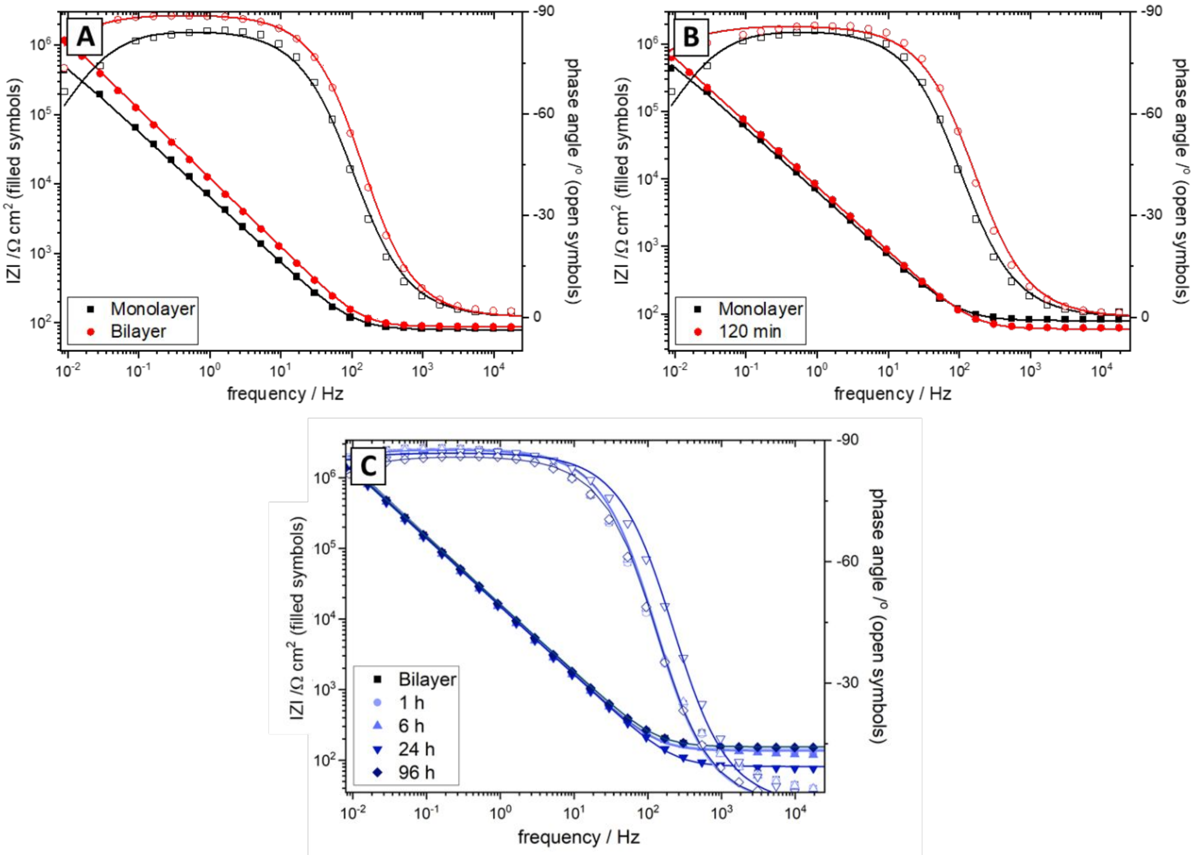

Figure S 5 A) Measurements of a mixed SAM formed in a solution containing 1 mM DPhyTL and 4 mM mercaptoethanol. B) addition of 1 mM ethyl hexanoate to the SAM. C) Bilayer stability over time. The full set of fitting data can be found in Table S 4 below. Symbols represent experimental data and lines represent the fit. The full set of fitting parameters and additional data can be found in Table S 1.

Table S 1: Fitting data of the Bode plots shown in . For clarity, we have omitted some of the data shown in the table below from the Bode plots shown in .

	Resistance (MΩ cm²)	Error (MΩ cm²)	Capacitance (µF/cm²)	Error (µF/cm²)	α
**Comparison of monolayer and bilayer**					
Monolayer	1.3	0.4	27.3	1.9	0.95
Bilayer	8.1	1.6	13.2	0.7	0.98
**Monolayer + 1 mM ethyl hexanoate over time**					
Monolaer	1.3	0.4	27.3	1.9	0.95
0 min	4.1	1.1	23.4	1.4	0.95
30 min	1.9	0.5	21.5	1.3	0.95
60 min	6.0	1.5	22.0	1.3	0.95
120 min	5.8	1.5	22.4	1.3	0.94
**Bilayer stability**					
Bilayer	56.0	28.2	10.4	0.8	0.98
1 h	54.2	27.3	10.5	0.8	0.98
6 h	51.4	39.0	10.8	0.8	0.98
24 h	52.6	23.9	11.3	0.7	0.98
96 h	20.6	5.7	10.7	0.5	0.98

Determination of frequency-independent membrane capacitance

Charging curves of the lipid bilayers (see Figure S 6) can be used to approximate membrane capacitance at a DC voltage by treating the system as a simple RC circuit in which the capacitance can be estimated based on the charging rate using Equation 1:

$$I_t = I_0 * \ e^{\frac{-t}{\tau}} \qquad \text{Equation 1}$$

Where I is the current at time t, I_0 is the current at t = 0, and τ is the time constant. The time constant is determined by the resistance and capacitance of the circuit:

$$\tau = RC \qquad \text{Equation 2}$$

The charging curves of the membranes are described relatively well by a simple exponential decay function (see Figure S 14A):

$$y = A_1 * \ e^{\left(\frac{-x}{t_1}\right)} + k \qquad \text{Equation 3}$$

in which t_l corresponds to τ. R can be calculated using V = I*R where R is the resistance of the sub-membrane reservoir and can be calculated from *k*, as this corresponds to the leakage current through the membrane defects and is limited by the resistance of the sub-membrane reservoir. At an applied transmembrane potential of V = 0.08 V and *k* = 1.76 * 10^{-12}, R = 4.5 * 10^{10} Ω (45 GΩ, a value in good agreement with the resistance determined by EIS, see Table S 6). For τ = 0.54 and using an electrode area of approximately 1000 µm2 (see Figure S 16B), this leads to a membrane capacitance of 1.2 µF/cm^2.

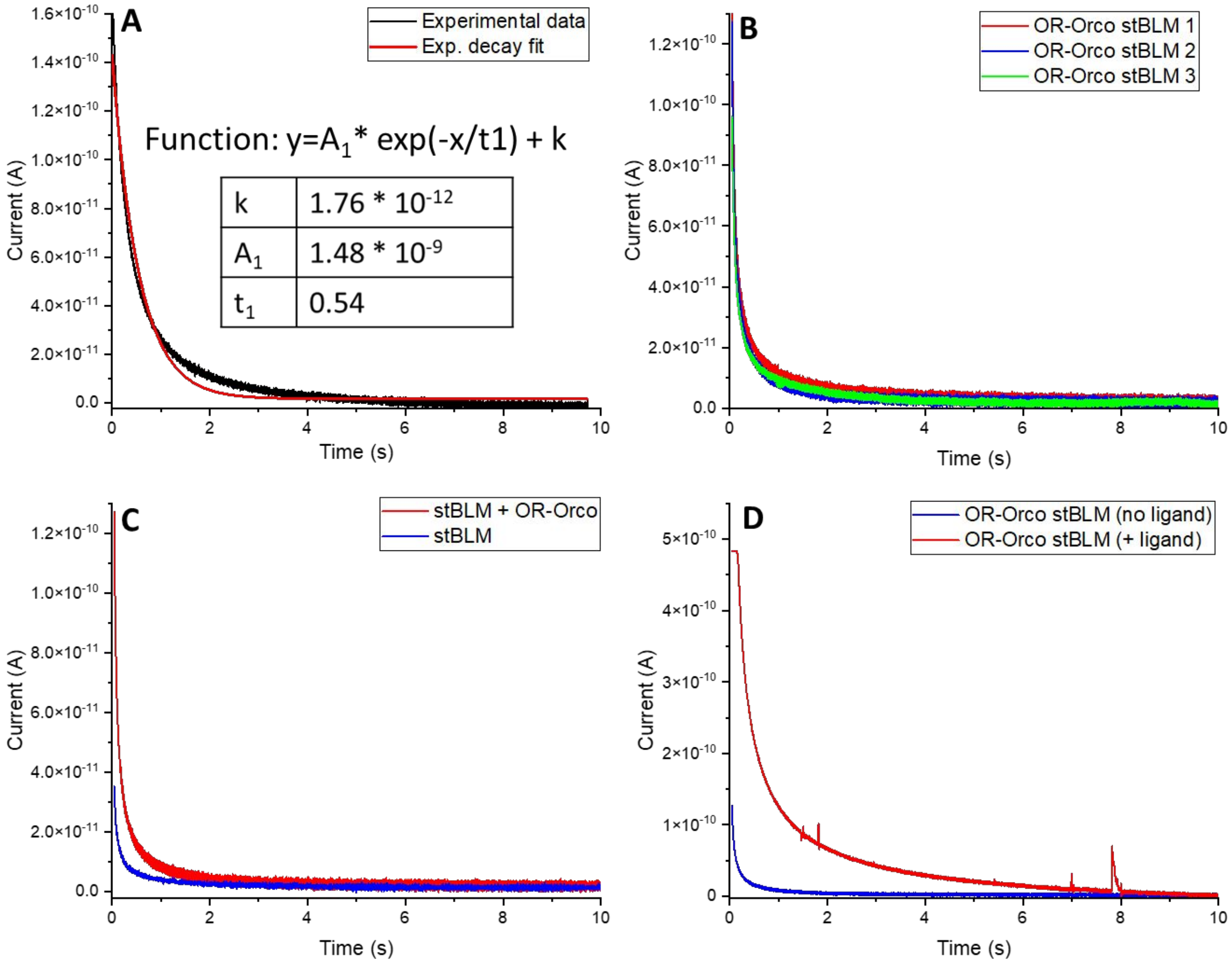

Figure S 6: Charging curves of stBLMs. A) OR-Orco sstBLM charging curve (black) and simulated exponential decay function describing capacitor charging (red). B) Additional replicates of charging curves on different OR-ORco sstBLMs. C) Comparison of charging curves of protein-free membrane (blue) and OR-Orco sstBLM (red). D) charging curves of OR-Orco sstBLM before and after addition of ethyl hexanoate (blue and red, respectively).

We repeated these calculations for several additional datasets or OR-Orco sstBLMs and protein-free membranes as well as before and after ligand addition, which allowed us to estimate the DC capacitances of these systems. The results are summarized in Table S 2. The capacitance after ligand addition is a combination of the capacitance of the open ion channels and that of the spacer and Helmholtz double layer.

Table S 2: Capacitance in µF/cm² estimated from the charging curves in Figure S 7. Each value is the average of three different datasets and the error is the standard deviation.

**OR-Orco (no ligand)**	**OR-Orco (+ ligand)**	**DPhyPC**
2.78 ± 0.61	26.15 ± 5.79	1.48 ± 0.15

Given that a simple exponential decay function is not an ideal fit, we expect that there is a larger deviation from the values reported in Table S 2 than the errors suggest, particularly given that the capacitance of protein-free membranes is overestimated by more than 30%. However, even taking this into account, a

capacitance of 2-3 μF/cm^2 is in excellent agreement with a membrane comprised of a lipid bilayer with a capacitance of 1 μF/cm^2 containing approximately 2% protein (estimated based on the AFM data of 8-16 receptors/μm^2 with a radius of 30 nm) using a dielectric constant of 60 which has been estimated for ion channels *via* simulations.[1]

Confirmation of bilayer formation by surface plasmon resonance

In order to confirm that the layer observed in SPR has the physicochemical properties expected of a lipid bilayer environment, we added the polarity-sensitive dye NileRed (NR) to the lipid membrane after formation (10 μM in 1X PBS with 1% DMSO, a DMSO concentration shown previously not to damage the lipid bilayer[2]). Nile red fluoresces strongly in non-polar environments such as organic solvents or lipid bilayers but is quenched in aqueous media.[3] Figure S7 and S8 show SPR and SPFS data.

As a reference, we used a stBLM with an inner leaflet comprised of 100% DPhyTL and an outer leaflet of DPhyPC (see supporting information Figure S6). We chose this membrane composition to reflect the "optimal" low-defect, high density state of a DPhyPC-based lipid membrane. To form protein-free lipid bilayers on the mixed SAMs used here, cholesterol must be included in the lipid mixture to form a lipid bilayer. This would not be a suitable protein-free analogue of the lipid bilayer, particularly as cholesterol affects Nile red fluorescence.[4]

A progressive increase in fluorescence was observed during dye incorporation followed by a reduction in fluorescence intensity upon rinsing with 1x PBS, after which a stable fluorescence level of 465 ± 43 cps was reached with an sstBLM containing OR22a-Orco. This is ~75% lower than the protein-free lipid membranes but still well above background fluorescence (see Figure S7B), indicating formation of a lipid bilayer. In addition to hindered dye uptake of protein-containing membranes, it is also possible that the presence of membrane proteins alters the dielectric properties of the membrane itself, reducing the fluorescence yield of the dye. NR resides at the membrane interface[4]Klicken oder tippen Sie hier, um Text einzugeben. near the headgroups, therefore we expect that all fluorescence we observed is due to dye located in the outer membrane leaflet. The small amounts of dye that could be located in the inner leaflet would be quenched due to the closer proximity of the gold film.

The decrease in fluorescence upon rinsing can be attributed to the removal of dye molecules not fully incorporated into the lipid bilayer. The increased fluorescence of protein-free membranes is expected, as there is significantly more space for dye incorporation when no proteins are present. However, this does not explain why the fluorescence in protein-containing membranes was 80% below the level of protein-free systems. As EIS experiments showed the dielectric constant of protein-containing membranes is higher than protein free-membranes, and the emission intensity of NR decreases exponentially with increasing dielectric constant of its environment.[5] Therefore, the local dielectric environment experienced by NR in protein-containing membranes differs from that of protein-free membranes.

As OR22a responds most strongly to ethyl hexanoate we chose this ligand to confirm correct receptor function. To ensure changes in membrane resistance can be attributed to ion channel opening rather than defect formation induced by ethyl hexanoate, we tested whether the addition of this compound at a high concentration changes the fluorescence level. This would mean a change in the polarity in close proximity to the dye, indicating the possibility of water-filled defects.

We introduced a high concentration of 100 μM ethyl hexanoate, as the formation of conductive defects which might decrease membrane resistance in a way that could be mistaken for ion channel opening would require the formation of water-filled defects. Formation of such defects would result in a decrease in fluorescence as the polarity of the dye environment increases, quenching fluorescence. However, fluorescence intensity remained steady in the presence of ethyl hexanoate (see S7B). Therefore, any reductions in membrane resistance can be attributed to ion channel activity. Given that membrane capacitance doubles upon ligand addition, some fluorescence quenching might be expected. However,

the loss in emission intensity resulting from a shift in dielectric constant from 10 to 40 is significantly less than the reduction observed below a dielectric constant of 5.[5]Klicken oder tippen Sie hier, um Text einzugeben. Moreover, only dyes that are already quenched by their proximity to the ion channel are affected which already have significantly reduced fluorescence. If defects were formed in protein-free areas of the lipid bilayer where fluorescence remains high, the reduction in fluorescence intensity would be much more significant.

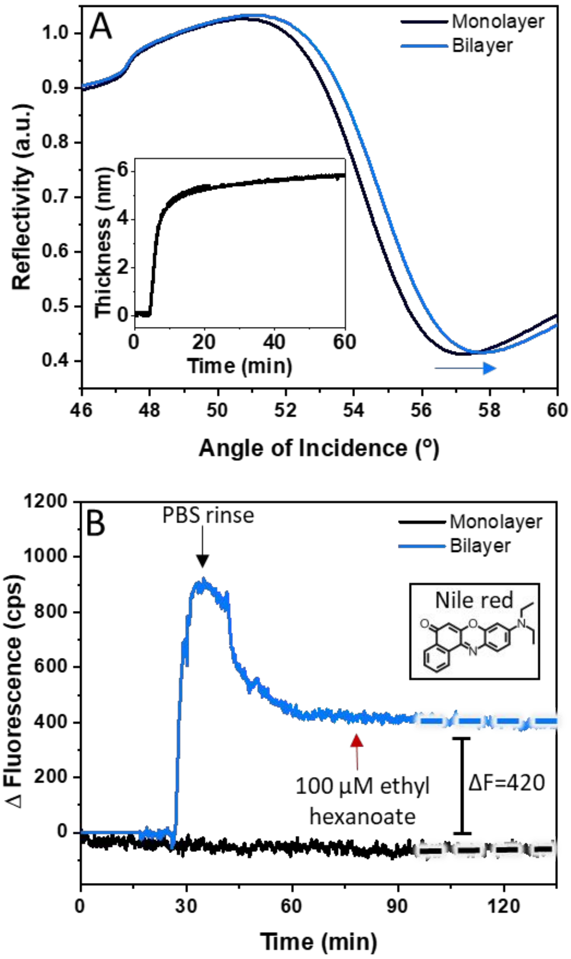

Figure S 7: A) Angular scan of an sstBLM containing OR22a/Orco (black), showing a layer thickness of 6 nm. The inset shows kinetics of bilayer formation. B) Fluorescence upon incubation of the membrane with the polarity-sensitive dye Nile Red (10 µM) over time and of the SAM prior to bilayer formation (black), The arrows indicate washing off the dye and the addition of 100 µM ethyl hexanoate (red and black, respectively). Additional data can be found in the supporting information (Figure S6).

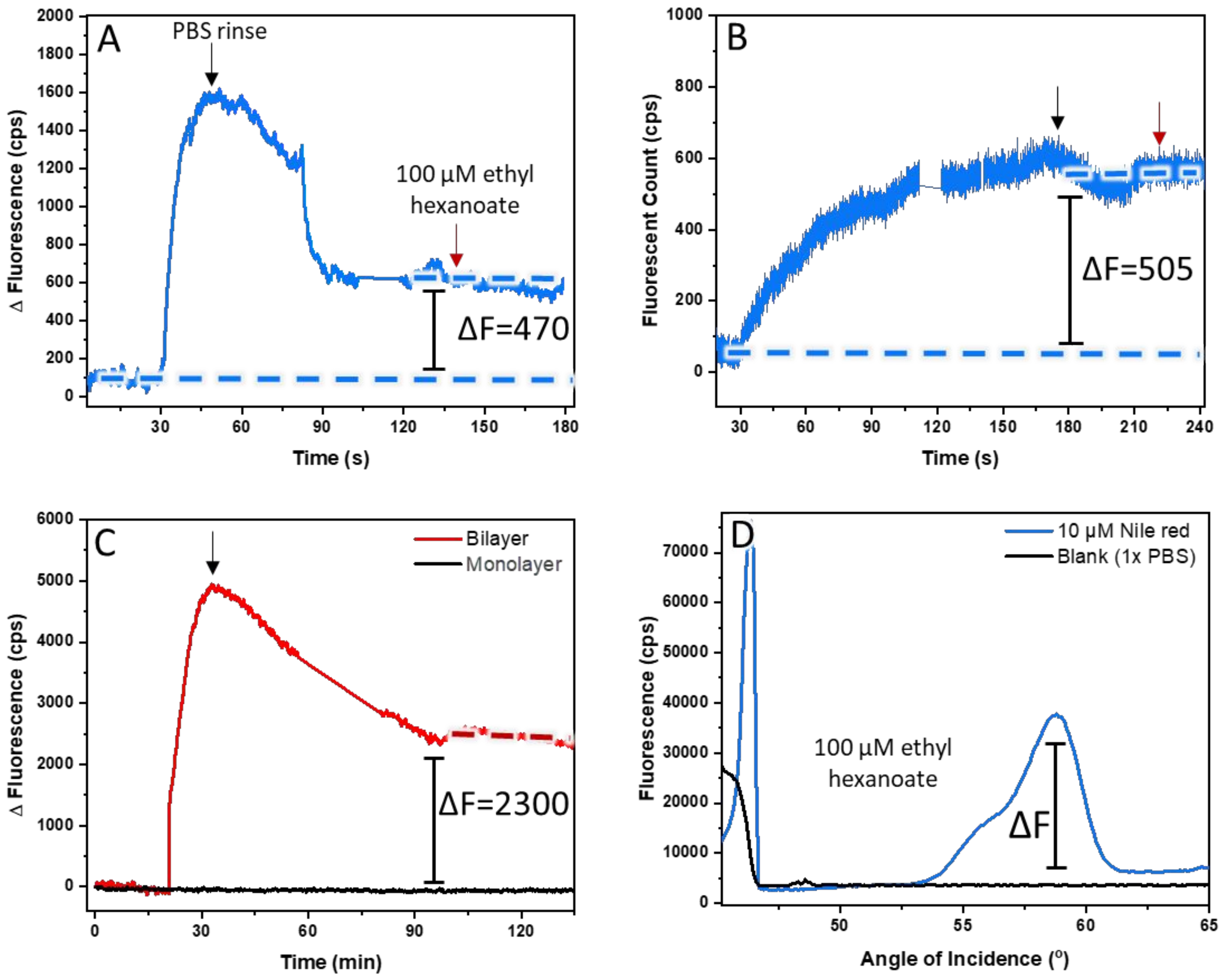

Figure S 8: A) and B) additional replicates of nile red addition to OR22a-Orco sstBLMs. C) incubation of protein-free sstBLM comprised of DPhyPC with 10 wt-% cholesterol. D) Angular scan of green laser used to induce fluorescence to determine the angle of maximum fluorescence.

The characteristic unbinding peak observed in A and the data shown in the main paper is much less pronounced in B, but after rinsing the same ΔF can be seen as in the other experiments. As we attribute this unbinding process to the removal of unfused vesicles and poorly incorporated dye molecules, the smaller unbinding peak likely indicates that there were fewer vesicles adsorbed to the bilayer prior to rinsing. Fitting parameters to determine layer thickness are shown in below.

Table S 3: fitted thickness parameters of an Orco-stBLM and adsorbed vesicles as determined by SPR.

**Layer**	**Fitted thickness (nm)**	**Refractive index (RIU)**
Or22a-Orco-stBLM		
Glass	infinite	1.845
Chromium	5.9	3.482
Gold	50	0.55
Lipid bilayer	5.8	1.48
Water (background)	infinite	1.33
Adsorbed vesicles		
Glass	Infinite	1.845
Chromium	5.9	3.482
Gold	50.0	0.55
Lipid bilayer	50.7	1.37
Water (background)	infinite	1.33

To estimate the refractive index of the adsorbed vesicles, a vesicle was assumed to be a two-layered sphere with the inner layer comprised of water (n = 1.33) with a diameter of 21 nm surrounded by a lipid bilayer (thickness 4 nm) with a refractive index of 1.45. Gold and epoxy glue thicknesses were measured with a dry scan and the resulting values used to fit the measurements in PBS.

In the kinetic observation of layer formation *via* surface plasmon spectroscopy, the SPR response was converted to refractive index changes and then to layer thickness by calibrating with a series of three known sucrose concentrations. Refractive index changes were calculated based on the calibration plot shown in Figure S 9.

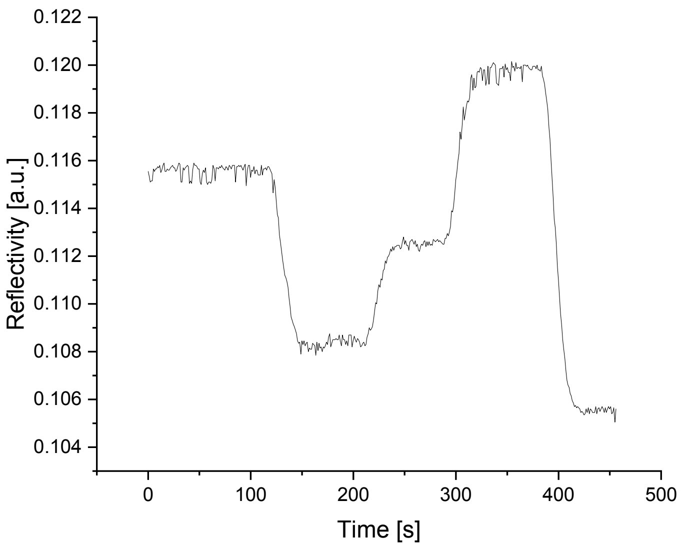

Figure S 9: Sucrose calibration curve with 1, 2 and 4 % sucrose solution respectively (starting from a 1x PBS solution baseline and flushing with water)

Confirmation of bilayer formation by Atomic force microscopy

To determine the density of the receptors, we filtered AFM data for grains with heights in the range of 0.8-1 nm above the membrane plane. Setting the maximum peak area what not straightforward, as the area of the features changes with scan rate and resolution. Peak area and height decreased with increased resolution and lower scan rates. As both the proteins and the lipid bilayers are flexible, they can easily be deformed by the AFM tip. The longer the tip is in contact with the protein, the more it is likely to be deformed.

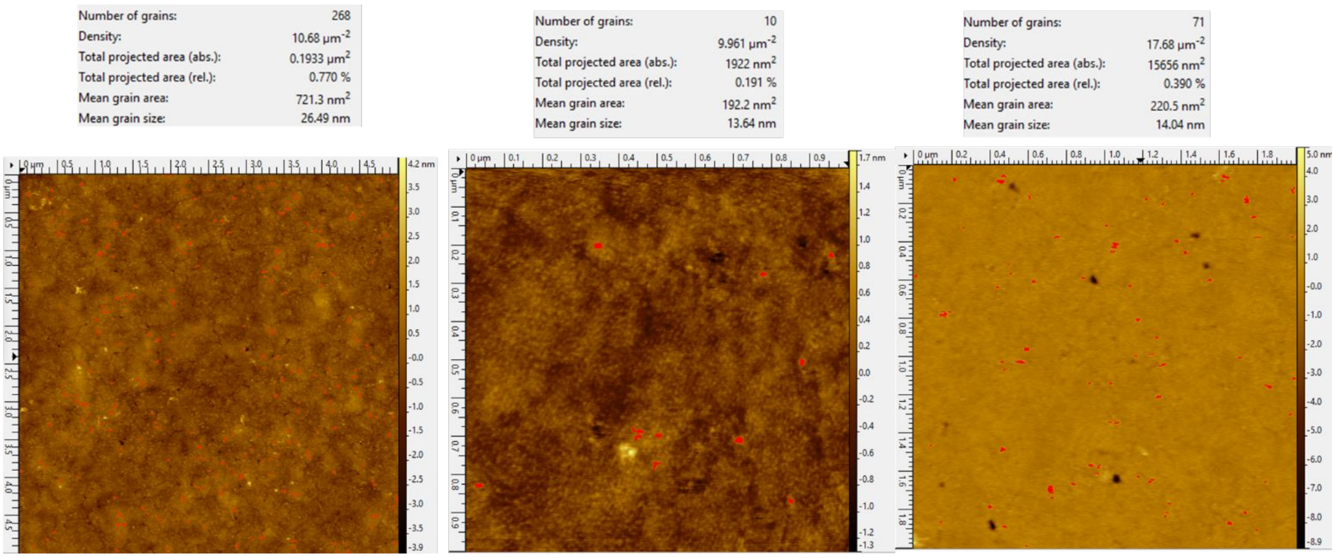

Figure S 10: AFM data used to determine ion channel density in the membranes based on finding peaks with a height of 0.8 – 1.2 nm. The points included in the statistical analysis are marked in red.

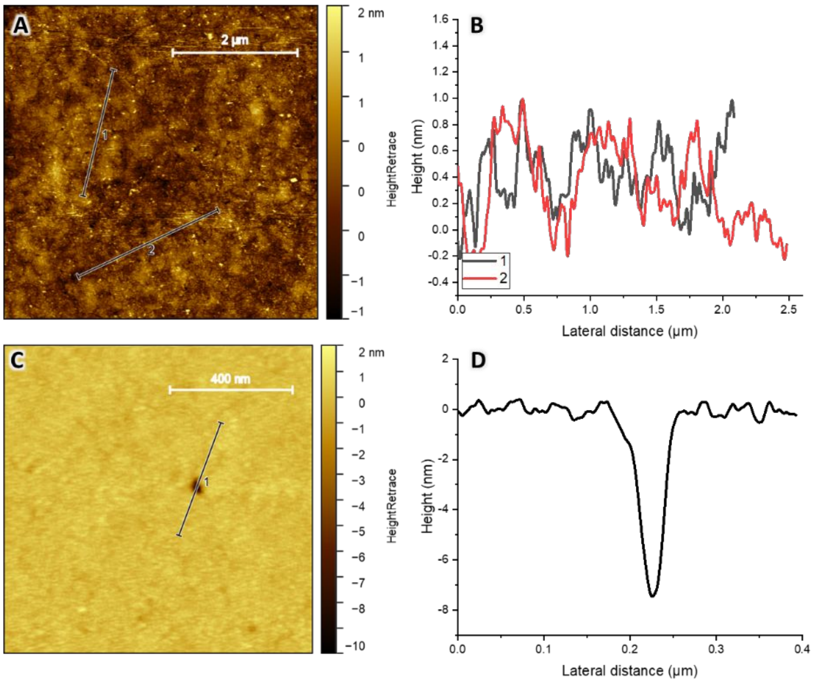

Figure S 11: A) additional AFM data and corresponding height traces (B) showing peaks with a height of around 1 nm. C) AFM image of bilayer with pinhole defect corresponding roughly to the height of a stBLM. D) height trace across the defect shown in C.

The depth of the defect shown in Figure S 12D shows the thickness of approximately 6 nm of the membrane architecture, demonstrating that sufficient space exists underneath the membrane to accommodate the sub-membrane domain of Orco (4 nm in total, accounting for the 2 nm thickness of the lipid bilayer. Additional defects with a depth between 5 and 7 nm are shown in

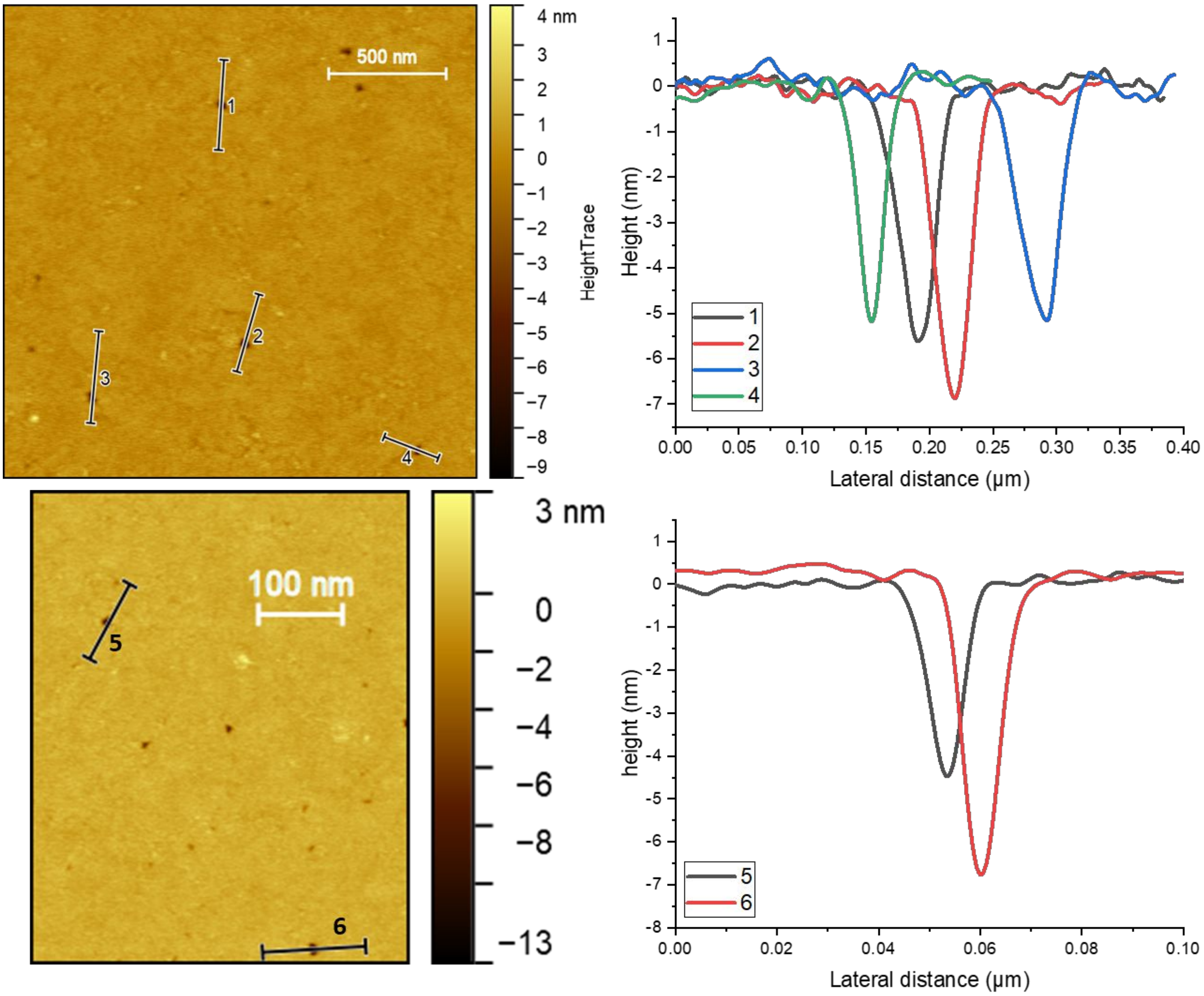

Figure S 12: AFM height traces of additional membrane defects.

Confirmation of receptor function by EIS

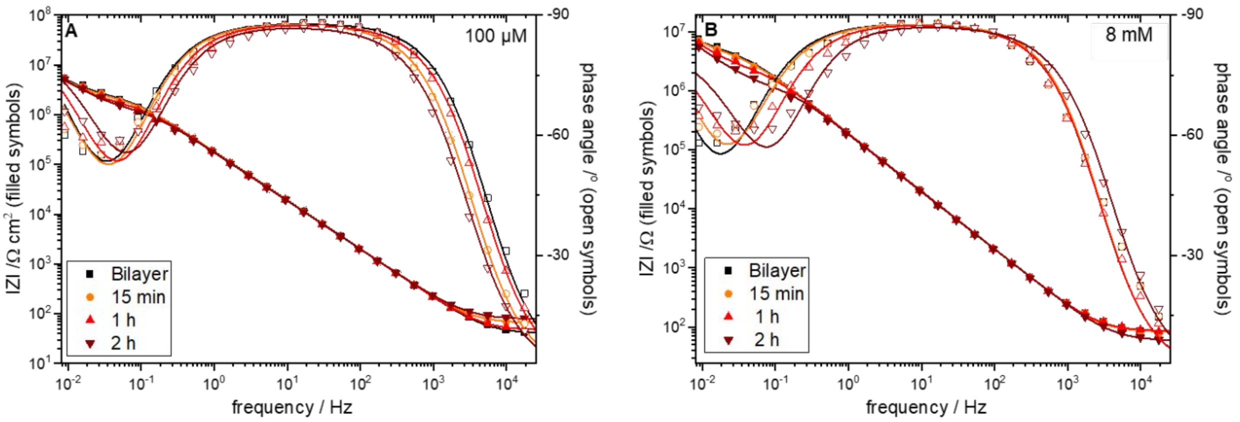

Figure S13: A) Addition of 100 µM ethyl hexanoate to protein-free tethered membranes comprised of DPhyPC on 100% DPhyTL. B) addition of 8 mM ethyl hexanoate to protein-free tethered membranes comprised of DPhyPC on 100% DPhyTL. Fitted data can be found in Table S 4.

For the control experiments, we used significantly higher ethyl hexanoate concentrations (8 mM, the approximate solubility limit of ethyl hexanoate in water) and still only observed very minor changes in membrane resistance even after two hours.

Table S 4: Fitting data of the Bode plots shown in Figure S13. For clarity, we have omitted some of the data shown in the table below from the Bode plots shown in Figure S14.

	Resistance (MΩ cm²)	Error (MΩ cm²)	Capacitance (µF/cm²)	Error (µF/cm²)	α
**Incubation of protein-free membrane with 100 µM ethyl hexanoate**					
Bilayer	1.9	0.4	1.2	0.1	0.98
15 min	1.6	0.2	1.2	0.1	0.98
1 h	1.2	0.2	1.3	0.1	0.97
2 h	0.9	0.1	1.4	0.1	0.98
**Incubation of protein-free membrane with 10 mM ethyl hexanoate**					
Bilayer	3.9	1.1	1.2	0.2	0.98
15 min	3.2	0.9	1.2	0.2	0.97
1 h	1.6	0.3	1.3	0.1	0.97
2 h	0.8	0.1	1.4	0.1	0.96

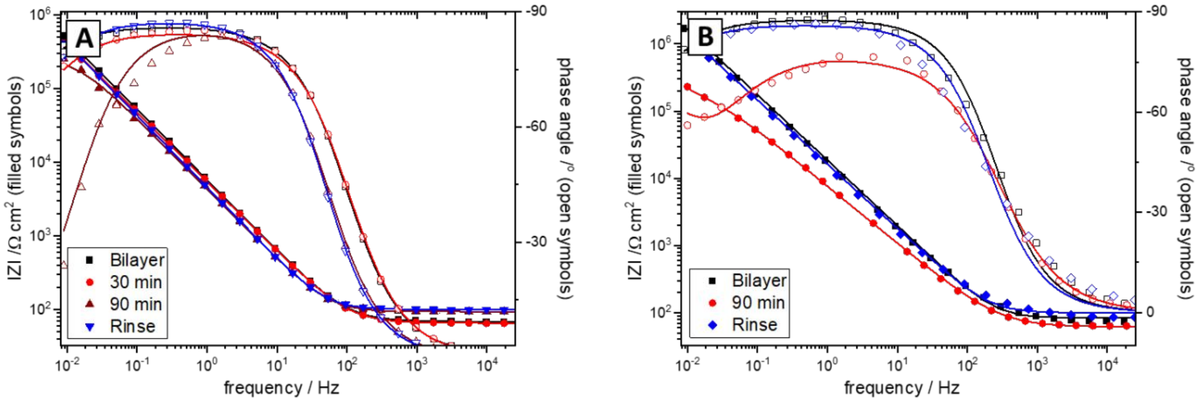

Figure S 14: A) Repeated addition of ethyl hexanoate to the stBLM shown in after rinsing. B) Additional data set of OR22a-Orco sstBLM incubated with 1 µM ethyl hexanoate over time followed by rinsing. The full set of fitting data can be found in Table S 5. Symbols represent experimental data and lines represent the fit.

Table S 5: Fitting parameters of Bode plots shown in Figure S 14. For clarity, we have omitted some of the data shown in the table below from the Bode plots shown above.

	Resistance (MΩ cm^2)	Error (MΩ cm^2)	Capacitance (µF/cm^2)	Error (µF/cm^2)	α
**Incubation of membrane with 1 µM ethyl hexanoate over time**					
0 min	13.7	2.6	12.0	0.1	0.98
30 min	10.2	1.7	12.5	0.1	0.97
60 min	8.63	1.32	13.0	0.1	0.94
90 min	0.08	0.004	62.3	1.0	0.85
120 min	0.58	0.05	50.8	0.4	0.87
150 min	0.82	0.09	42.7	0.3	0.89
180 min	0.80	0.09	43.2	0.3	0.88
Rinse	12.0	3.1	12.0	0.1	0.98
**Repeated addition of 1 µM ethyl hexanoate after rinse**					
Rinse (after first ligand addition)	12.0	3.1	12.0	0.1	0.98
10 min	5.2	0.5	30.0	0.1	0.98
30 min	2.6	0.2	32.8	0.2	0.97
90 min	0.3	0.0	39.8	0.7	0.88
Rinse	2.6	0.2	36.5	0.2	0.98
**Addition of 1 µM ethyl hexanoate (replicate on new bilayer)**					
Bilayer	11.0	2.3	9.0	0.4	0.98
120 min	0.14	0.04	35.9	8.2	0.87
Rinse	10.6	5.6	10.6	0.8	0.97

Calculation of ion channel density:

By saturating the membrane with 100 µM ethyl hexanoate, which has been shown to produce the maximum response of Or22a/Orco,[7] we assume that all active ion channels inserted in the correct orientation and their native structures will open. We can therefore assume that the resistance of the membrane after incubation with 100 µM ethyl hexanoate is a combination of the resistance of the membrane containing no ion channels and the ion channels themselves. Based on the structure of the Orco-pore,[7] we assume an ion channel diameter of 1 nm and a single channel conductivity of 2.5 pA at a potential of 80 mV.

$$Conductivity = \frac{1}{R} = \frac{I}{V}$$ Equation 4

R=3.2*10^{10} Ω (31 pS)

Assuming an ion channel diameter of 1 nm, $A_{ion\ channel} = 7.8*10^{-15}\ cm^2$

$$R_{norm} = R * A_{ion\ channel} = 3.2 * 10^{10}\ \Omega * 7.8 * 10^{-15} \frac{cm^2}{ion\ channel} = 0.00025 \frac{\Omega\ cm^2}{ion\ channel}$$ Equation 5

Total resistance of all ion channels R_{total} = 58 kΩ

$$Ion\ channel\ density = \frac{R_{total}}{R_{norm}} = \frac{58000\ \Omega}{0.00025\ \Omega\ cm^2} = \frac{2.3\ x\ 10^8}{cm^2}$$ Equation 6

At a resistance of 58 kΩ cm^2 after ligand addition, we can calculate a receptor density of approximately 2.3 receptors per μm^2. Using the other datasets of 140 and 64 58 kΩ cm^2, we estimate a range of 2.3-5.5 receptors/μm^2.

Single channel measurements

Preparation of electrodes: Fabricating micrometre-sized electrodes which allow combination with the template stripping process was done by grating a micropattern into a 50 nm deposition layer of Au on Si-wafer. An xy-translational stage was used to form a rectangular electrode which was observed under a light microscope. Once the electrode was formed, Teflon tape was fixed onto the surface again with the help of a light microscope to further reduce the effective surface area, as seen in Figure S 19.

Statistical analysis: single-channel activity has been analyzed using all-point current histograms.[8] The all-point current histograms shown in Figure 6 summarize the measured current for all sample points starting at time 1.5. The bin size is chosen as 0.1 pA. To limit the plot area, all bars below 5% of the maximum bar are excluded. For the control experiments, the raw data was additionally denoised by subtracting the baseline, which was obtained by fitting an exponential regression to the data starting at time 2. The all-point histogram was plotted on the resulting data. Amplitude distributions for the histograms were then fitted with two gaussian components. For this, the histogram data was first smoothed using the Savitzky-Golay filter and the peaks of the resulting curve were calculated. Using the largest peak as centre, the first gaussian component was fitted. After subtracting the resulting distribution from the histogram, the second component was fitted to the remaining data. Finally, the histogram has been shifted such that the larger of the two gaussian distributions is centred at 0. The distributions represent the opening (smaller peak) and closing (higher peak) of the channel. The distance of the means of the two distributions corresponds to the current level of the opened channel.

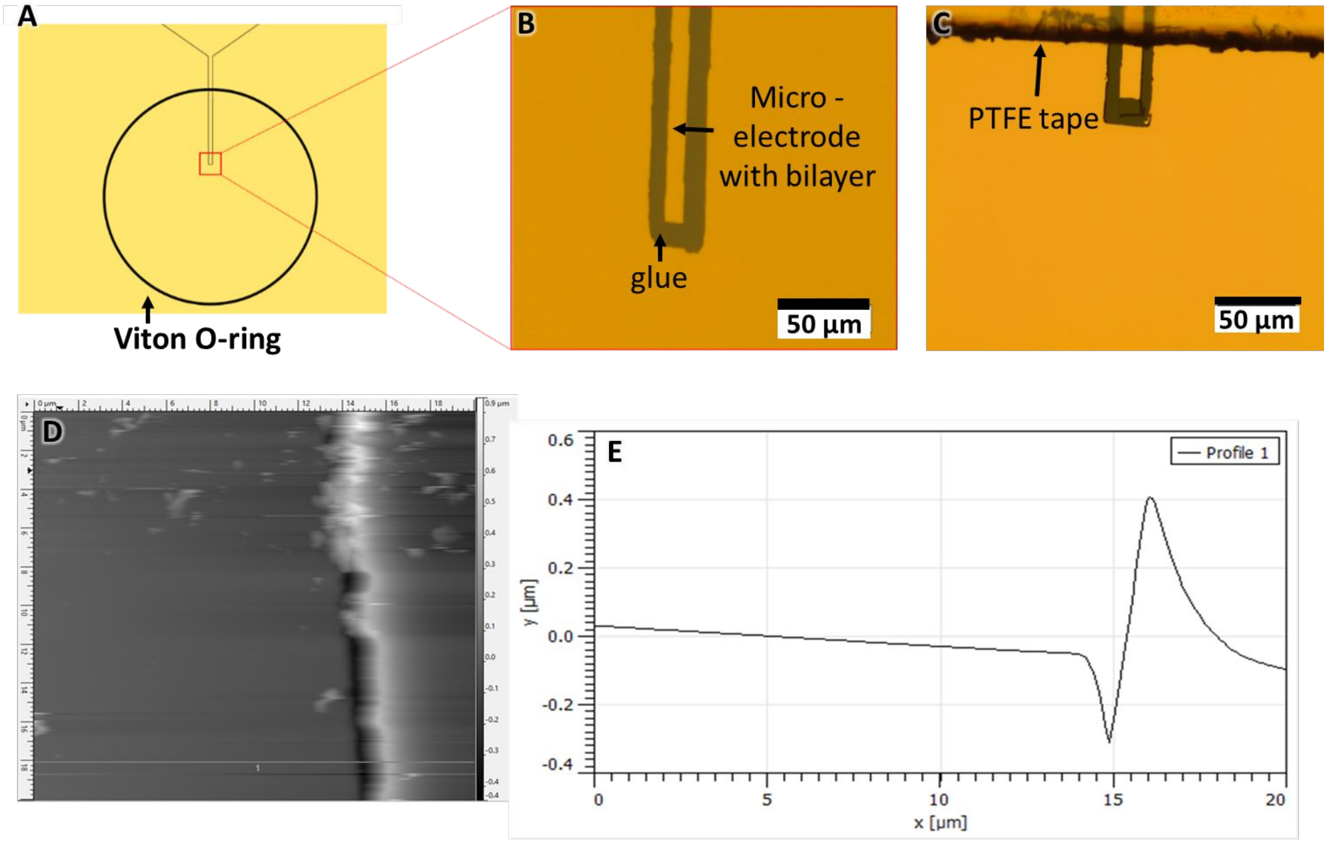

Figure S 15: A) measurement setup for single channel and EIS experiments on microelectrodes. B) optical microscope image of microelectrode. C) reduction of electrode area with PTFE tape. D) AFM image of the transition from the microelectrode to the glue, showing some possible defects created during the template stripping process. E) height trace across D).

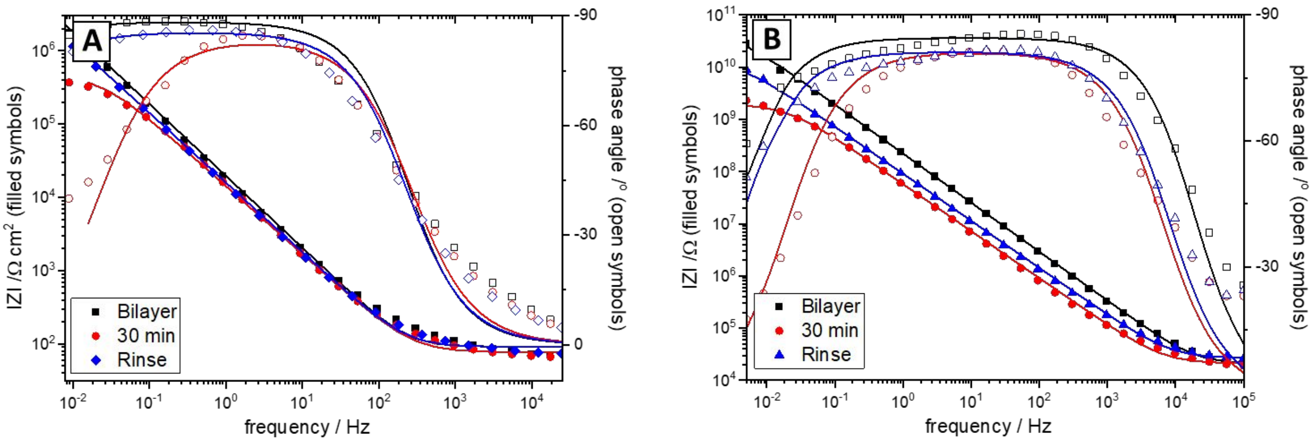

Figure S 16: A) Addition of 2 µM EcorOBP-m1 incubated with 10 nM ethyl hexanoate. B) Addition of 1 µM ethyl hexanoate to OR22a-Orco sstBLM formed on microelectrodes used for single channel recording. The electrode area in B) was 1000 µm². Full set of fitting data can be found in Table S 6. Symbols represent experimental data and lines represent the fit.

Table S 6: Fitting parameters of Bode plots shown in Figure S17. For clarity, we have omitted some of the data shown in the table below from the Bode plots shown above.

	Resistance (GΩ)	Error (GΩ)	Capacitance (nF)	Error (nF)	α
**Addition of 1 µM ethyl hexanoate to membrane formed on microelectrode**					
Bilayer	45.09	7.49	0.81	0.03	0.99
60 min	1.98	0.16	3.30	0.17	0.92
Rinse	13.00	2.37	2.09	0.09	0.95
	Resistance (MΩ cm^2)	Error (MΩ cm^2)	Capacitance (µF/cm^2)	Error (µF/cm^2)	
**Addition of 10 nM ethyl hexanoate with 2 µM EcorOBP15-m1**					
Bilayer	50.9	22.1	8.5	0.7	0.98
30 min	0.48	0.08	12.6	0.9	0.87
Rinse	15.1	7.5	10.9	0.8	0.96

Please note that the data of the measurements made on the microelectrodes (first dataset shown in Figure S 20A and Table S 6, as only the absolute resistance is relevant for single channel recordings.

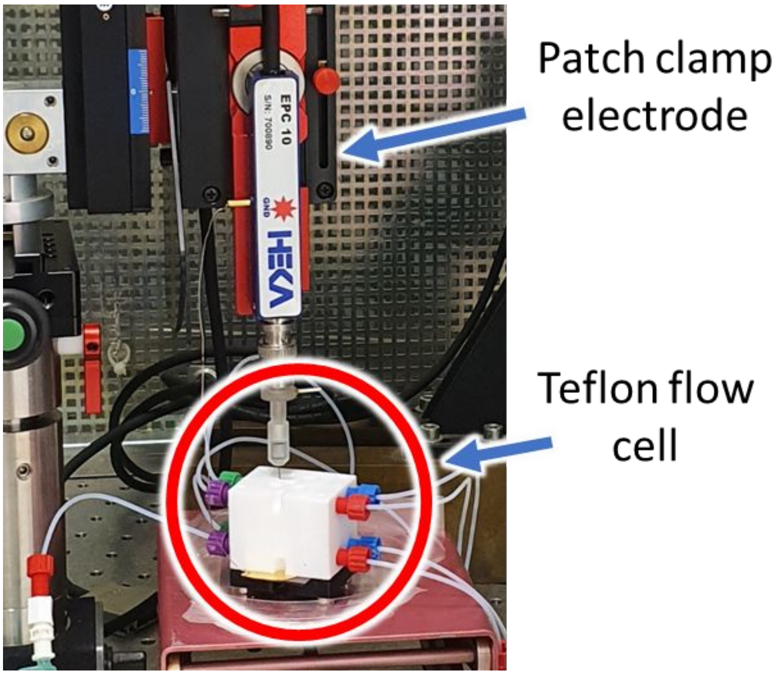

Figure S 17: Setup used for single channel measurements inside a double faraday cage.

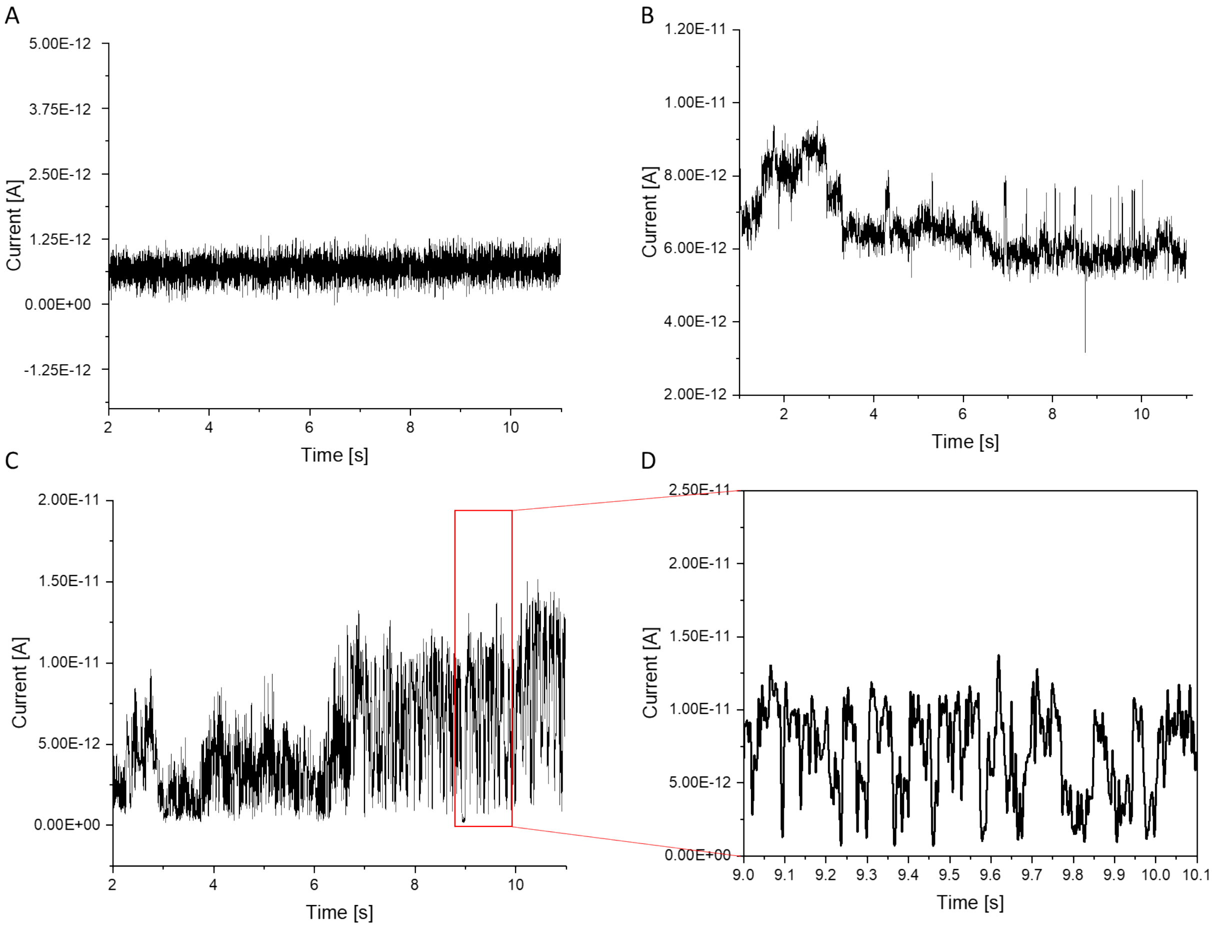

Figure S18: Tip-dip recordings of OR22a-Orco in DPhyPC bilayers. A) recording prior to addition of ethyl hexanoate in 1x PBS, 80 mV, B) OR22a-Orco activity upon addition of 100 µM ethyl hexanoate and 1 µM OBP14-m1 at 80 mV, C) additional measurement of OR22a-Orco activity upon addition of 100 µM ethyl hexanoate and 1 µM OBP14-m1 at 80 mV and D) close-up view of the highlighted section of C.

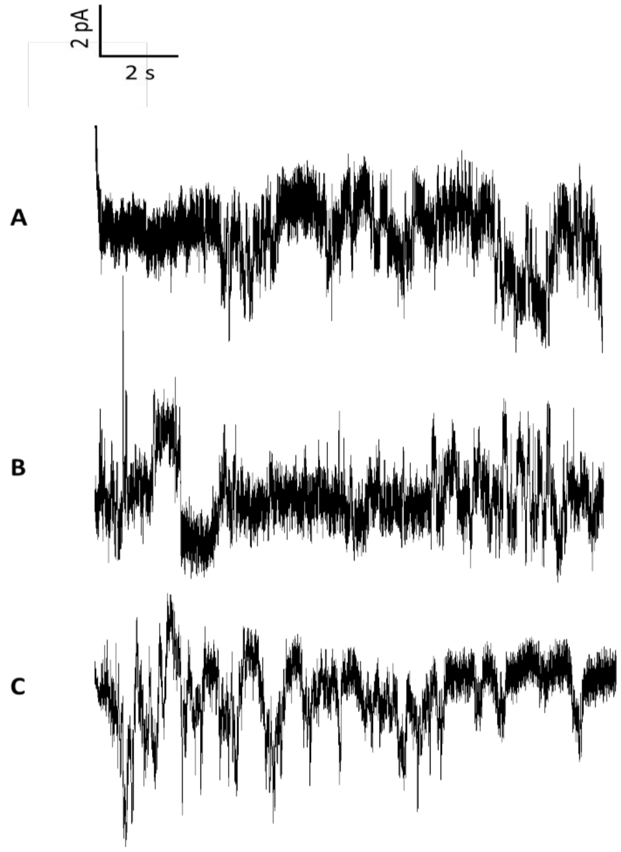

Figure S 19: Additional opening events of Or22a-Orco triggered by adding 100µM hexanoate solubilized by EcorOBP15-m1. A-C represent independent measurements under the same conditions

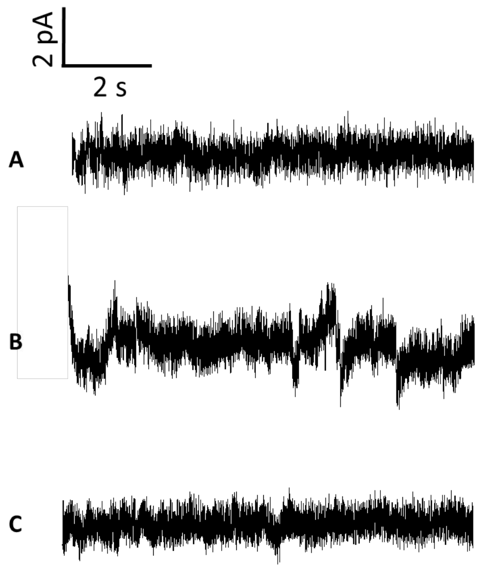

Figure S 20: PBS blank (A), opening triggered by ethyl hexanoate plus OBP (B), PBS flush after opening (C)

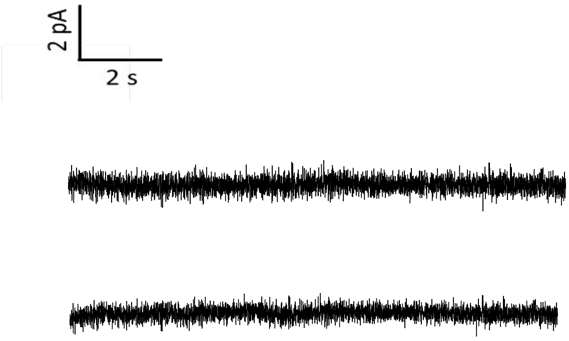

Figure S 21: Response of receptor-free stBLMs to addition of 100 µM ethyl hexanoate. Membranes are comprised of DPhyPC with 20 wt-% percent cholesterol.

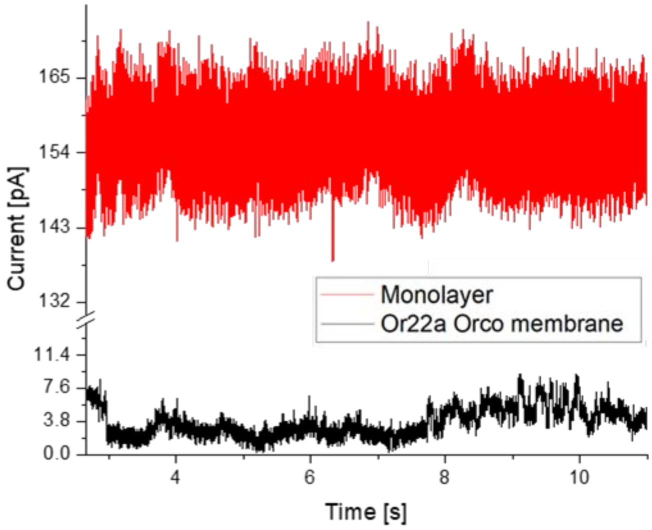

Figure S 22: Patch clamping measurement on SAM with ethyl hexanoate only showing background current (red) significantly exceeding what would be necessary to observe single channel activity (black). A background current of 2 pA at an applied voltage of 80 mV indicates a membrane resistance of approximately 40 GΩ.

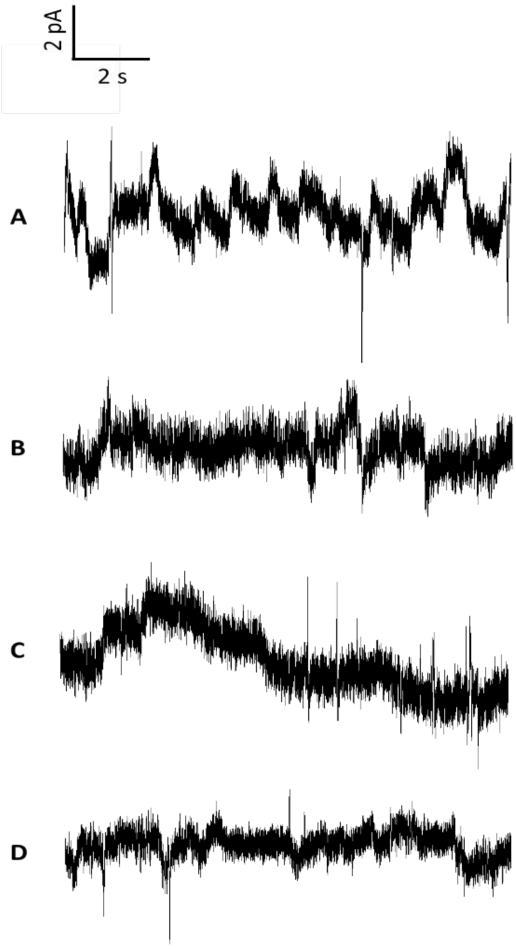

Figure S 23: Additional opening events of Or22a-Orco triggered by gaseous ethyl hexanoate solubilized by EcorOBP15-m1. A-D represent independent measurements under the same conditions.

References

1. Baştuğ, T.; Kuyucak, S. Role of the Dielectric Constants of Membrane Proteins and Channel Water in Ion Permeation. *Biophysical Journal* 2003, 84 (5), 2871–2882. https://www.sciencedirect.com/science/article/pii/S0006349503700150.
2. Kleinheinz, D.; D'Onofrio, C.; Carraher, C.; Ramach, U.; Schuster, B.; Bozdogan, A.; Knoll, W.; Andersson, J. Functional Incorporation of the Insect Odorant Receptor Coreceptor in Tethered Lipid Bilayer Nanoarchitectures. *Biosensors and Bioelectronics* 2022, 203, 114024. https://www.sciencedirect.com/science/article/pii/S0956566322000641.
3. Greenspan, P.; Fowler, S. D. Spectrofluorometric Studies of the Lipid Probe, Nile Red. *Journal of Lipid Research* 1985, 26 (7), 781–789. DOI: 10.1016/S0022-2275(20)34307-8.
4. Mukherjee, S.; Raghuraman, H.; Chattopadhyay, A. Membrane Localization and Dynamics of Nile Red: Effect of Cholesterol. *Biochimica et biophysica acta* 2007, 1768 (1), 59–66. DOI: 10.1016/j.bbamem.2006.07.010.
5. Hess, C. M.; Riley, E. A.; Reid, P. J. Dielectric Dependence of Single-Molecule Photoluminescence Intermittency: Nile Red in Poly(vinylidene fluoride). *The journal of physical chemistry. B* 2014, 118 (29), 8905–8913. DOI: 10.1021/jp505874m.
6. Münch, D.; Galizia, C. G. DoOR 2.0--Comprehensive Mapping of Drosophila melanogaster Odorant Responses. *Scientific Reports* 2016, 6, 21841. DOI: 10.1038/srep21841.
7. Butterwick, J. A.; del Mármol, J.; Kim, K. H.; Kahlson, M. A.; Rogow, J. A.; Walz, T.; Ruta, V. Cryo-EM Structure of the Insect Olfactory Receptor Orco. *Nature*, 2018, 447–452.
8. Encyclopedia of Biophysics. Patch-Clamp Recording of Single Channel Activity: Acquisition and Analysis; Noel Wyn Davies, Ed.; Springer, 2013.
